# Genetic mapping and genomic selection for maize stalk strength

**DOI:** 10.1186/s12870-020-2270-4

**Published:** 2020-05-07

**Authors:** Xiaogang Liu, Xiaojiao Hu, Kun Li, Zhifang Liu, Yujin Wu, Hongwu Wang, Changling Huang

**Affiliations:** Institute of Crop Sciences, National Key Facility of Crop Gene Resources and Genetic Improvement, Chinese Academy of Agricultural Sciences, Beijing, 100081 China

**Keywords:** Stalk strength, Rind penetrometer resistance, Quantitative trait loci, Genomic selection, Maize

## Abstract

**Background:**

Maize is one of the most important staple crops and is widely grown throughout the world. Stalk lodging can cause enormous yield losses in maize production. However, rind penetrometer resistance (RPR), which is recognized as a reliable measurement to evaluate stalk strength, has been shown to be efficient and useful for improving stalk lodging-resistance. Linkage mapping is an acknowledged approach for exploring the genetic architecture of target traits. In addition, genomic selection (GS) using whole genome markers enhances selection efficiency for genetically complex traits. In the present study, two recombinant inbred line (RIL) populations were utilized to dissect the genetic basis of RPR, which was evaluated in seven growth stages.

**Results:**

The optimal stages to measure stalk strength are the silking phase and stages after silking. A total of 66 and 45 quantitative trait loci (QTL) were identified in each RIL population. Several potential candidate genes were predicted according to the maize gene annotation database and were closely associated with the biosynthesis of cell wall components. Moreover, analysis of gene ontology (GO) enrichment and Kyoto Encyclopedia of Genes and Genomes (KEGG) pathway further indicated that genes related to cell wall formation were involved in the determination of RPR. In addition, a multivariate model of genomic selection efficiently improved the prediction accuracy relative to a univariate model and a model considering RPR-relevant loci as fixed effects.

**Conclusions:**

The genetic architecture of RPR is highly genetically complex. Multiple minor effect QTL are jointly involved in controlling phenotypic variation in RPR. Several pleiotropic QTL identified in multiple stages may contain reliable genes and can be used to develop functional markers for improving the selection efficiency of stalk strength. The application of genomic selection to RPR may be a promising approach to accelerate breeding process for improving stalk strength and enhancing lodging-resistance.

## Background

Stalk lodging can seriously influence photosynthesis and substance transportation, and annually causes reductions in maize yield ranging from 5 to 20% worldwide [1]. Several factors, such as genetics, natural conditions, field management, diseases and insect pests, can result in weak plant standability and stalk lodging [2–6]. Strong stalks can reduce the occurrence of lodging because stalk mechanical strength is negatively correlated with stalk lodging [7–12]. Hence, stalk strength can be used to evaluate stalk lodging-resistance. Several approaches have been developed to measure stalk strength, including stalk bending strength, stalk crushing strength, and rind penetrometer strength (RPR) [13–18]. Compared to other methods, RPR has obvious advantages in terms of its simple and efficient operation and no requirement for destroying the structure of the stalk and impacting plant growth [1, 14, 15, 19–21]. Consequently, investigating RPR in multiple growth stages can be viewed as an available and feasible strategy to determine the optimal measurement period. In addition, RPR has been significantly and positively correlated with stalk lodging-resistance in previous studies [9, 14, 15, 22]. Moreover, purposeful selection for RPR has been performed and has been shown to be able to simultaneously improve stalk quality and lodging-resistance [8, 15, 23–25].

As for the genetic architecture of RPR, previous studies have used association and linkage mapping to identify quantitative trait loci (QTL) with the purpose of developing functional markers to carry out marker-assisted selection (MAS) to improve stalk strength. Specifically, multiple F_2:3_ populations genotyped by simple sequence repeats (SSR) markers have been used to dissect the genetic basis of RPR, and then to compare the efficiency of phenotypic selection and MAS. A total of 35 QTL have been detected that corresponded with RPR, which clearly implies the complex nature of stalk strength [1, 21]. Moreover, MAS for high RPR has been shown to be more effective than phenotypic selection when the QTL are derived from the same population rather than from separate populations [23]. In addition, several recombinant inbred line (RIL) and double haploid (DH) populations have been constructed to explore RPR-related loci, and high-quality linkage maps have been established using single nucleotide polymorphism (SNP) markers. The potential candidate genes predicted from these studies are directly or indirectly associated with the biosynthesis of lignin and cellulose, illustrating that cell wall components are likely involved in the determination and formation of RPR [16, 19, 20, 26, 27]. On the other hand, a range of significantly associated loci related to RPR have been detected in genome-wide association study (GWAS) for maize nested association mapping panel and natural population, further indicating the great genetic complexity of RPR [28, 29]. Despite that, genomic selection (GS) for stalk strength has rarely been reported, and unsatisfactory prediction accuracies have been obtained in previous studies [16, 28]. The predictive ability needs to be improved to be able to predict stalk strength in a GS strategy for practical breeding programs.

In this study, we focus on dissecting the genetic architecture of RPR and providing several useful pieces of advice for plant breeders to improve the selection efficiency for stalk lodging-resistance. The datasets in the present study that contained phenotypic data that are evaluated in seven stages in two RIL populations and genotypic data obtained from SNP array were used to identify RPR-relevant loci and perform genomic selection. Our objectives were to (1) ascertain the optimal measure stage for stalk strength; (2) dissect the genetic architecture of RPR; (3) predict potential candidate genes and biological pathway associated to RPR; and (4) perform genomic selection by using multiple models to enhance breeding efficiency and seeking a few advisable measures for practical breeding schemes.

## Results

### Phenotypic variation and complex relationship between stages in RPR

**Fig. 1:**
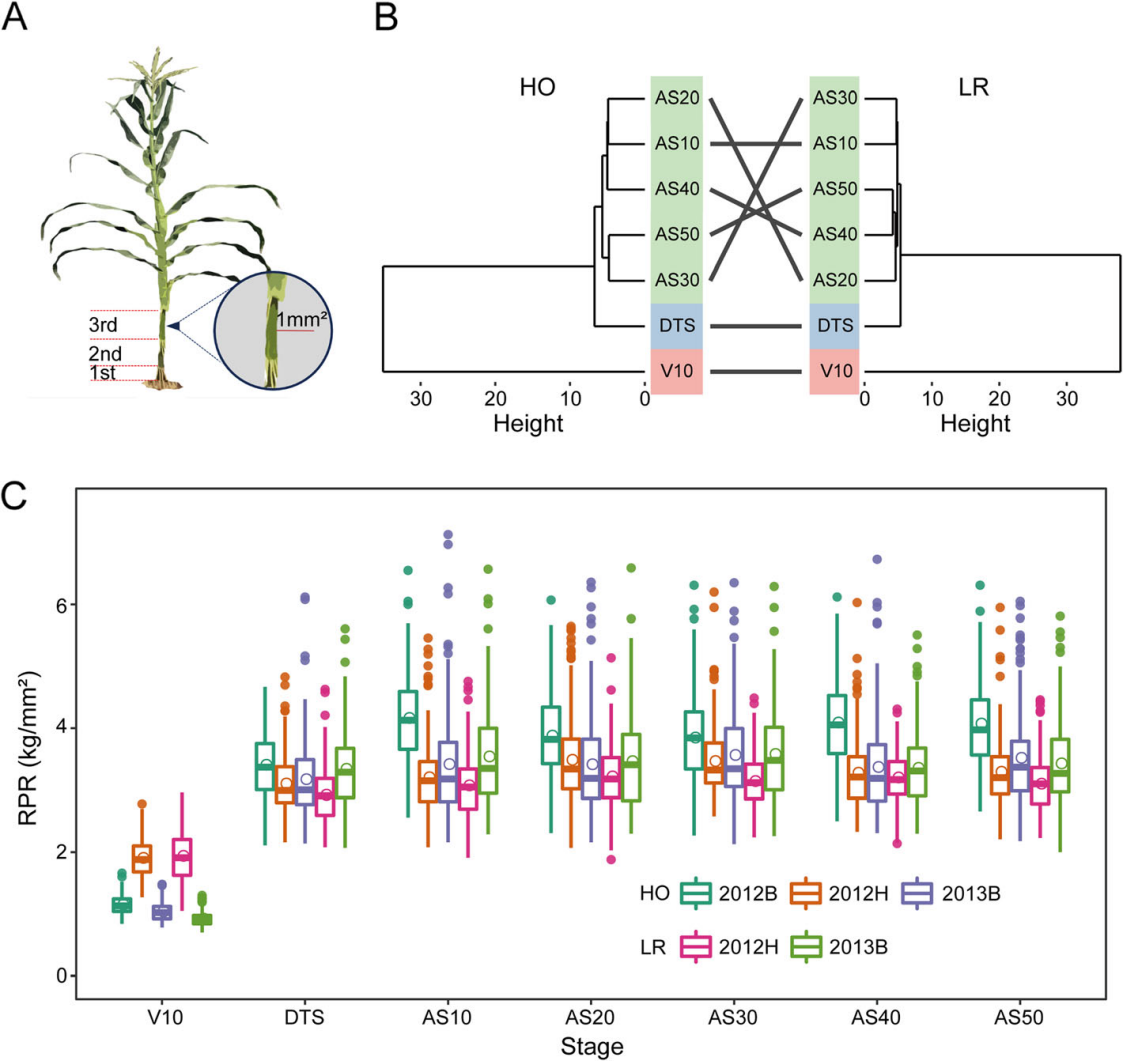
Extensive phenotypic variation of rind penetrometer resistance in each RIL population. **a** Diagram of the RPR measurement. The cross-sectional area of the probe is 1 mm^2^. **b** Hierarchical clustering of RPR evaluated in seven stages in each RIL population. Height is defined as the Euclidean distance between clusters. **c** Phenotypic variation of RPR measured in seven stages across multiple environments. HO: the high-oil population (B73 × BY804); LR: the lodging-resistance population (Zheng58 × HD568); RPR: rind penetrometer resistance; V10: the tenth-leaf stage; DTS: days to silking; AS10: 10 days after silking; AS20: 20 days after silking; AF30: 30 days after silking; AS40: 40 days after silking; AS50: 50 days after silking; 2012B: Beijing in 2012; 2012H: Hainan in 2012; 2013B: Beijing in 2013

According to previous studies and research results from our group [6, 10, 11, 30], the third stalk internode above the ground was selected to investigate RPR to assess stalk strength (Fig. 1a). The RPR values evaluated in multiple stages, environments and RIL populations exhibited normal distributions, and the difference between the smallest and largest values ranged from 1.90- to 3.33-fold for each stage (Additional file 1: Figure S1). Additionally, the phenotypic correlation coefficients between V10 and the other stages were lower than those between DTS and the stages after silking and much lower than *r*_*p*_ between stages after silking (Additional file 1: Figure S1). A phenotypic clustering analysis was performed following the phenotypic variation of RPR in two RIL populations (Fig. 1b). The RPR values in the seven stages were classified into three groups, and the largest cluster consisted of AS10, AS20, AS30, AS40 and AS50, which belong to the reproductive growth phase. Stages V10 and DTS were separately classified into other two groups (Fig. 1b). On the other hand, the boxplot of RPR of each RIL population was drawn using data from various stages across environments. More precisely, RPR increased from stage V10 to stage AS50 in each environment, and then a slight enhancement was obtained ranging from stage DTS to subsequent stages (Fig. 1c). In addition, RPR in stage V10 in 2012H was remarkably higher than that in stage V10 in other environments. Moreover, RPR evaluated from stage DTS to stage AS50 within 2012B was larger than that in other environments in both RIL populations (Fig. 1c). Analysis of variance was performed to estimate the broad-sense heritability. The *H*^*2*^ of RPR in stage V10 in each population was lowest relative to the other stages. The broad-sense heritability varied from stage to stage and was larger than 67.0% in all stages except stage V10 (Additional file 1: Table S1).

### Construction and quality of high-density linkage map

**Fig. 2:**
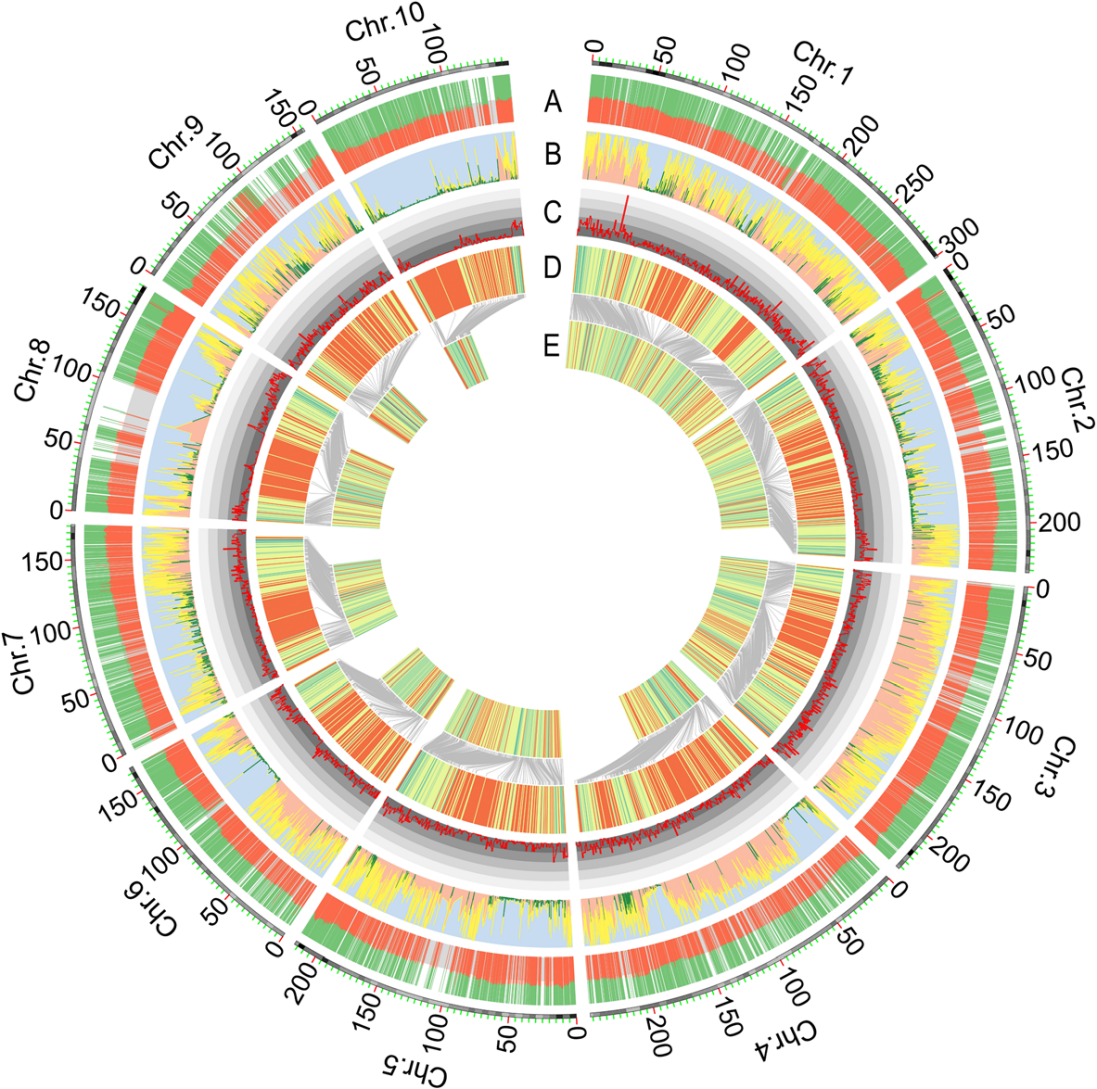
Distribution of and analysis process for genotypic data in the LR (Zheng58 × HD568) population. The outmost layer with the scale represents ten chromosomes in maize. **a** Distributed proportion of each polymorphic SNP within the RIL population. The red color represents the ratio of individuals with genotypes derived from Zheng58; green color represents the ratio of individuals with genotypes derived from HD568; gray color is the reference line for 0.5. **b** Distribution of chi-square values for each SNP within ten chromosomes. The green color denotes unqualified markers with *P* values lower than 0.05. **c** Density of qualified SNPs based on the chi-square test (1.0 Mb window size). The scale with different colors is an indicator of the number of markers within the unit window size, and the numbers from inner to outer are 0 to 50 with spacing of 10. **d** Physical distribution of bin markers on each chromosome. **e** Distribution of bin markers in the linkage map. Gray lines denote the comparison of the order of bin markers shared between physical and genetic maps

**Fig. 3:**
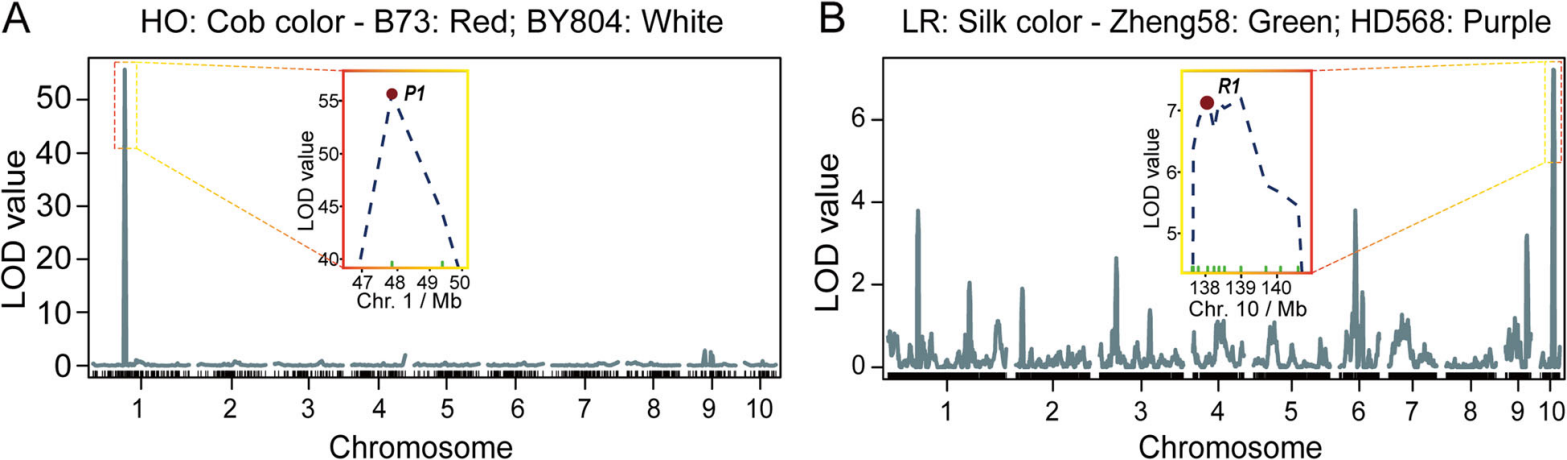
Mapping of *P1* and *R1* in each RIL population. **a** QTL mapping of cob color in the HO population (B73 × BY804). The red dot denotes the relative physical position of the *P1* gene. **b** QTL mapping of silk color in the LR population (Zheng58 × HD568). The red dot denotes the relative physical position of the *R1* gene

A total of 15,167 SNPs were retained after quality control for genotypic data, and then a total of 11,691 SNPs passed the chi-square test with a significance level over 0.05 in the LR population (Fig. 2). A sliding-window approach was applied to assign bin markers, and a set of 2121 recombination bins were used to construct the genetic map. The average physical length of the bin markers was 971 Kb, with a minimal length of 5.1 Kb and a maximal length of 54.4 Mb (Additional file 1: Table S2). There were 19 bin markers with a length of more than 10 Mb, and a total of 15 bins were located at centromeric or pericentromeric regions distributed in ten chromosomes (Additional file 1: Table S3). The other four recombination bins with a long physical length, including lmk0203, lmk0979, lmk1002, and lmk1453, were in regions with a lower SNP coverage because most of the SNPs in these regions were disqualified by chi-square tests (Fig. 2, Additional file 1: Table S3). Regarding the analysis of genotypic data in the HO population, a total of 756 SNPs were retained without segregation distortion (chi-square test, *P* > 0.05), and then these markers were used to construct genetic map and perform further analyses. The linkage maps for the HO and LR populations were constructed by the R package. The map lengths were 1642.2 and 1519.5 cM for each RIL population, respectively. Moreover, the average genetic lengths between adjacent markers were 2.2 and 0.7 cM for the HO and LR populations, respectively, which were equivalent to approximately 2.6 and 0.97 Mb in physical length (Additional file 1: Table S4). To evaluate the quality of the linkage maps, plots were drawn to compare the order of markers, which illustrated an excellent collinearity between physical and genetic maps (Fig. 2, Additional file 1: Figure S2). In addition, QTL mapping of cob color in the HO population and silk color in the LR population were performed to assess the power and accuracy of the genetic maps of each population. The QTL for cob and silk color, called *pC1* and *pS10* here, respectively, were detected with the highest LOD values of 55.7 and 7.2 for the peaks located at 47.8 and 138.1 Mb on chromosome 1 and 10, respectively (Fig. 3). For *pC1*, a cloned gene, *P1* (for *pericarp color1*), which conditions red flavonoid pigment and phlobaphene in the floral organs, including the kernel pericarp, cob glumes, tassel glumes, and silk [31, 32], lies in this QTL. A classical gene, *R1*, located in the QTL *pS1*0 that regulates anthocyanin pigmentation in tissues is corelated with the color of the kernel pericarp and silk [33, 34].

### QTL mapping of RPR in each RIL population

**Fig. 4:**
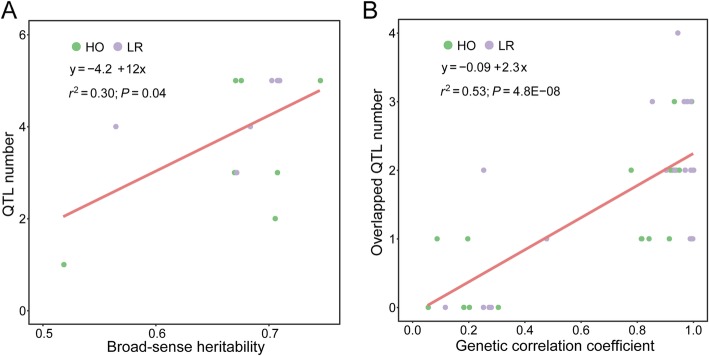
Relationship between QTL mapping and genetic parameters. **a** Relationship between QTL number and broad-sense heritability. **b** Relationship between overlapped QTL number and genetic correlation between all pairs of stages. HO: the high-oil population (B73 × BY804); LR: the lodging-resistance population (Zheng58 × HD568)

**Fig. 5:**
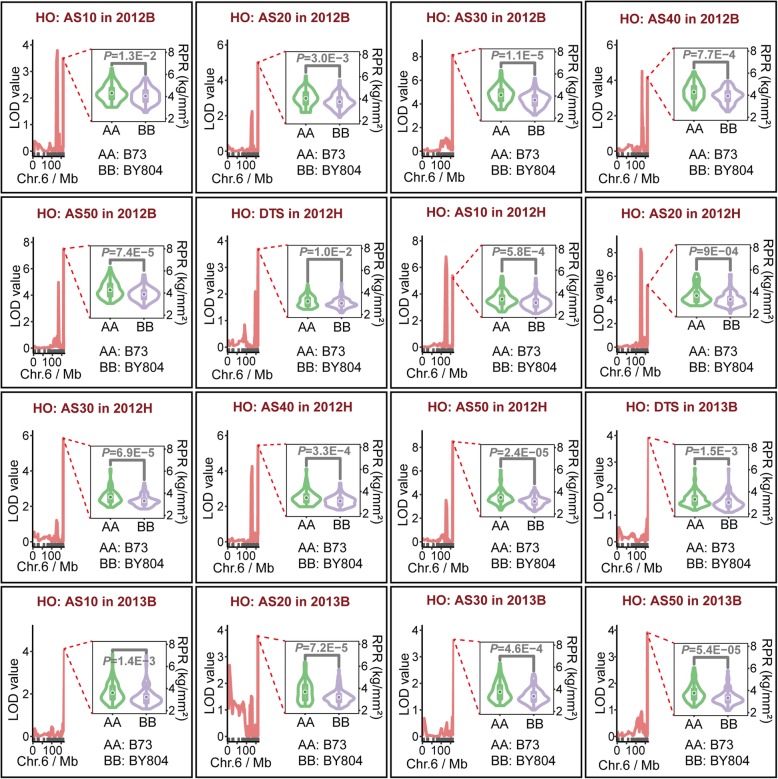
Illustration of pQTL6–2 identified in various situations. Violin plots denote the difference between genotypes derived from each parent; HO: the high-oil population (B73 × BY804); RPR: rind penetrometer resistance; DTS: days to silking; AS10: 10 days after silking; AS20: 20 days after silking; AF30: 30 days after silking; AS40: 40 days after silking; AS50: 50 days after silking; 2012B: Beijing in 2012; 2012H: Hainan in 2012; 2013B: Beijing in 2013

QTL mapping of RPR evaluated in seven stages in each environment was performed using a high-quality genetic map in the R package *R/qtl* version 1.44–9 [35]. A total of 66 QTL were detected for RPR in the HO population, which included 26, 20, and 20 QTL identified in the three environments. The physical lengths of the confidence intervals for these QTL spanned from 0.83 to 29.11 Mb, with an average length of 6.54 Mb. The phenotypic variance of RPR explained by each QTL ranged from 1.95 to 13.61%, with a mean value of 7.01% of the variation in RPR. The genetic effects for each QTL were estimated ranging from − 0.24 to 0.29 (Additional file 1: Table S5). Furthermore, the alleles derived from BY804 decreased the stalk strength in RPR when the value of the genetic effect was negative. On the other hand, alleles from B73 improved RPR when the genetic effect of the QTL was positive. A total of nine QTL could individually explain more than 10% of the phenotypic variance in RPR, and four of these QTL were detected in stage AS30 (Additional file 1: Table S5). In addition, a total of three QTL were identified in 2013B with overlapped confidence intervals and had higher genetic effects, indicating this QTL could improve RPR from 0.25 to 0.29 kg/mm^2^ (Additional file 1: Table S5). As for the LR population, a total of 45 QTL were identified for RPR in the two environments, which contained 23 and 22 QTL. The confidence intervals for each QTL spanned physical lengths from 0.70 to 64.49 Mb, with an average length of 8.37 Mb; 38 of these were shorter than 14.0 Mb in physical length (Additional file 1: Table S6). The phenotypic variance explained by each QTL for RPR ranged from 1.85 to 14.06%, with a mean value of 6.31% of the variation in RPR. Moreover, the genetic effects of each QTL were calculated ranging from − 0.19 to 0.26 (Additional file 1: Table S6). Besides, if the value of the genetic effect was positive, the alleles could derive from HD568 and enhance stalk strength in RPR. However, alleles could exert a negative effect on RPR when the genetic effect of QTL was negative (Additional file 1: Table S6). There were three QTL related to RPR that could singly explain more than 10% of the phenotypic variance, and 2 of these QTL were identified in stage AS10 (Additional file 1: Table S6). The relationship between the QTL number and broad-sense heritability was analyzed using BLUE values to perform QTL mapping in each population. The number of QTL increased as the broad-sense heritability estimated for RPR in each stage increased across environments (Fig. 4a). On the other hand, as the genetic correlation coefficient between stages increased, the number of overlapped QTL for both stages increased (Fig. 4b). Finally, a total of 18 pleiotropic QTL (pQTL) were detected by integrating the overlapped genomic region among 111 QTL for RPR in two populations, which were located on chromosomes 1 to 9 (Table 1). In particular, pQTL6–2, whose confidence interval encompassed 16 QTL that were identified in the HO population across three environments, was located on chromosome 6, with a physical length of 8.56 Mb. The phenotypic variance explained by this pQTL in different situations ranged from 3.57 to 13.31% of variation in RPR, with a mean of 7.81%. Furthermore, the genetic effects were estimated ranging from − 0.09 to − 0.24, with an average effect of − 0.19 (Table 1, Fig. 5, Additional file 1: Table S5). In addition, pQTL8 was identified and had a physical length of 22.42 Mb that included 9 QTL located on chromosome 8, of which eight QTL were derived from the LR population and another QTL was detected with a lower genetic effect in the HO population (Table 1, Additional file 1: Figure S3, Additional file 1: Table S5, S6).

**Table 1:** Pleiotropic QTL (pQTL) for rind penetrometer resistance in two RIL populations

pQTL^a^	Chr.^b^	Interval^c^ (Mb)	Physical length^d^ (Mb)	No. of QTL	Integrated QTL^e^
pQTL1–1	1	17.74–19.7	1.96	4	*qAhb1–1*, *qAhf1*, *qChb1*, *qChg1*
pQTL1–2	1	49.39–58.5	9.11	5	*qAhe1*, *qAhg1*, *qChc1*, *qChd1*, *qChe1–1*
pQTL1–3	1	212.31–234.79	22.48	3	*qBlf1–1*, *qCla1*, *qBhg1*
pQTL1–4	1	260.15–276.99	16.84	3	*qCha1–2*, *qChe1–2*, *qChf1*
pQTL2–1	2	22.97–31.29	8.32	4	*qAhb2*, *qAhc2*, *qAhd2*, *qChb2*
pQTL2–2	2	156.86–174.98	18.12	2	*qAhg2*, *qAhe2*
pQTL3–1	3	210.76–215.02	4.26	3	*qChe3*, *qChd3*, *qChf3*
pQTL3–2	3	224.13–225.51	1.38	3	*qClf3*, *qClg3*, *qClb3–2*
pQTL4–1	4	8.9–12.03	3.13	4	*qBlb4–1*, *qBlc4*, *qCld4*, *qCle4*
pQTL4–2	4	177.15–189.04	11.89	5	*qBhg4*, *qBla4*, *qBlf4*, *qClb4*, *qBlb4–2*
pQTL5–1	5	46.08–69.55	23.47	3	*qCle5*, *qClf5–1*, *qClg5*
pQTL5–2	5	208.1–210.4	2.3	4	*qClb5*, *qClf5–2*, *qClc5*, *qCld5–2*
pQTL6–1	6	118.15–137.9	19.75	7	*qBhc6–1*, *qAhc6–1*, *qBhd6–1*, *qBhf6–1*, *qBhg6–1*, *qAhf6–1*, *qAhg6–1*
pQTL6–2	6	158.47–167.03	8.56	16	*qAhe6*, *qAhg6–2*, *qAhc6–2*, *qAhd6*, *qAhf6–2*, *qBhb6*, *qBhc6–2*, *qBhd6–2*, *qBhe6*, *qBhf6–2*, *qChd6*, *qChg6*, *qBhg6–2*, *qChb6*, *qChc6*, *qChe6*
pQTL7–1	7	1.3–4.06	2.76	2	*qAhe7*, *qAhg7*
pQTL7–2	7	152.95–157.9	4.95	2	*qCla7–2*, *qBhb7*
pQTL8	8	118.33–140.75	22.42	9	*qClf8*, *qBlb8*, *qBlc8*, *qBle8–2*, *qBlg8*, *qClc8*, *qCle8*, *qBld8*, *qAha8*
pQTL9	9	129.19–133.92	4.73	2	*qAhc9*, *qAhd9*

^a^ The name of pleiotropic QTL

^b^ Chr.: number of chromosomes

^c^ Interval: physical range of flanking markers

^d^ Physical length: physical distance between flanking markers

^e^ The name of each QTL consists of information regarding the environment (A for 2012B; B for 2012H; C for 2013B), stage (a for V10; b for DTS; c for AS10; d for AS20; e for AS30; f for AS40; g for AS50), population type (h for high-oil population; l for lodging-resistance population), and number of the chromosome. V10: the tenth-leaf stage; DTS: days to silking; AS10: 10 days after silking; AS20: 20 days after silking; AF30: 30 days after silking; AS40: 40 days after silking; AS50: 50 days after silking. 2012B: Beijing in 2012; 2012H: Hainan in 2012; 2013B: Beijing in 2013

### GO enrichment and KEGG pathway analysis for candidate genes

**Fig. 6:**
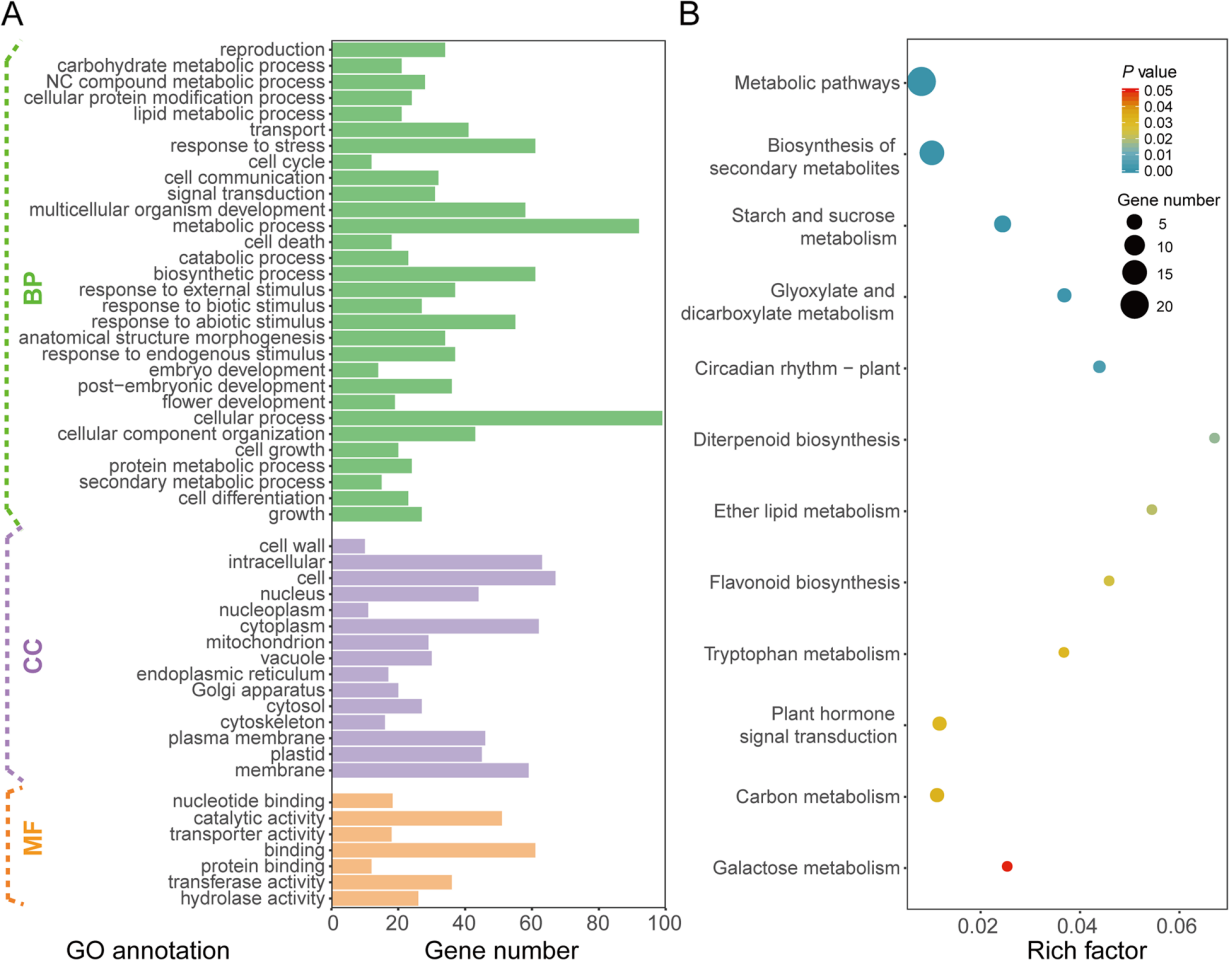
Analysis of GO enrichment and KEGG pathway based on predicted candidate genes. **a** Enrichment analysis of GO items. MF: molecular function related to candidate genes; CC: cellular component corresponding to candidate genes; BP: biological processes associated with candidate genes. **b** Analysis of KEGG pathway based on candidate genes. The color of the dot refers to the corrected *P* value; the size of the dot denotes the number of candidate genes in the pathway; the rich factor represents the ratio of the number of candidate genes in the pathway

According to the available database for maize gene annotation accessible at MaizeGDB, a total of 106 predicted candidate genes with physical regions corresponding to the confidence intervals of these QTL were selected and annotated. Moreover, these candidate genes were determined according to the list of classical genes described in the annotation database, and the description of biological function for the predicted genes was usually related to substance transportation and cell growth (Additional file 1: Table S7). GO analysis of the candidate genes illustrated that the enrichment items mainly included biological processes related to metabolism, biosynthesis, response to stress, and material transportation. In addition, the cell components relevant to genes consisted of the plasma membrane, Golgi apparatus, endoplasmic reticulum, and cell wall (Fig. 6a). As for the KEGG analysis of the predicted genes, a total of 12 pathways were identified (Fig. 6b). These pathways included the biosynthesis of secondary metabolites, starch and sucrose metabolism, plant hormone signal transduction, galactose metabolism, etc., which could be related to the formation of the cell wall and could contribute to the formation of RPR.

### Improving genomic selection for RPR using models considering fixed effects or multivariate

**Fig. 7:**
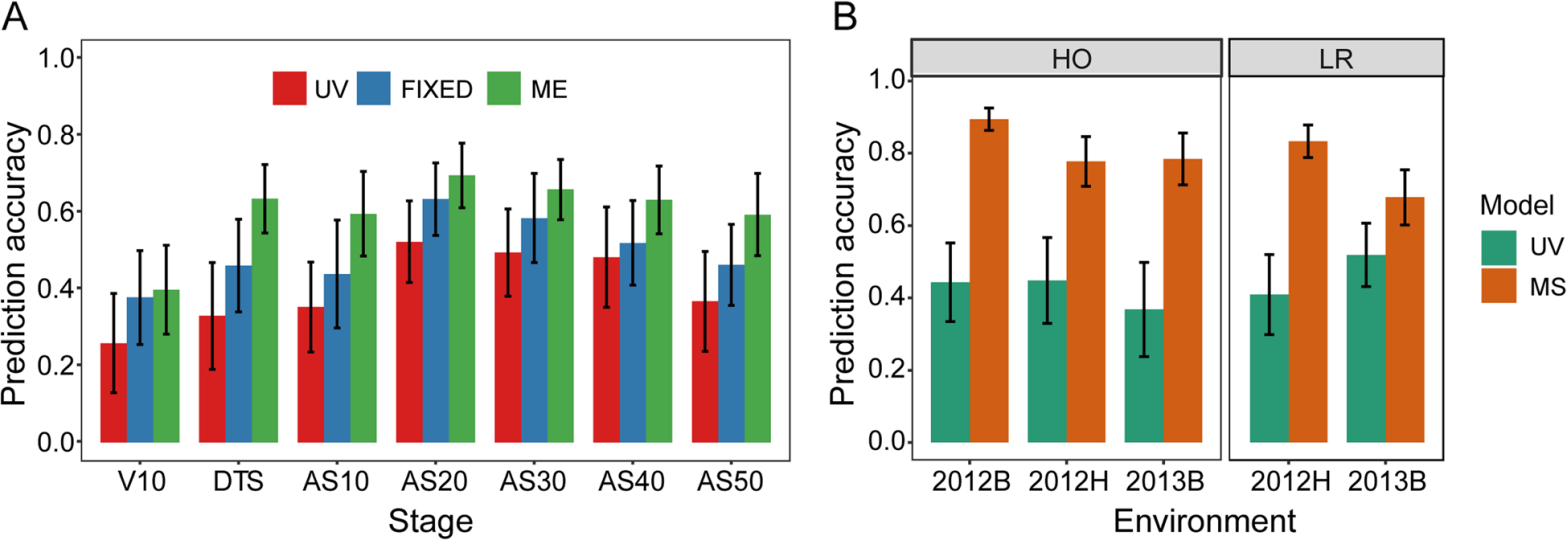
Comparison of the prediction accuracies between models. **a** Comparison of predictive ability between UV, FIXED and ME models in HO population. **b** Comparison of predictive ability between UV and MS models in two RIL populations. UV: univariate model, namely the general GBLUP model; FIXED: the GBLUP model considering RPR-relevant QTL as fixed effects; ME: multivariate GBLUP model using phenotypic data evaluated in other environments as auxiliary variates; MS: multivariate GBLUP model using phenotypic data evaluated in other stages as auxiliary variates; HO: the high-oil population (B73 × BY804); LR: the lodging-resistance population (Zheng58 × HD568); RPR: rind penetrometer resistance; V10: the tenth-leaf stage; DTS: days to silking; AS10: 10 days after silking; AS20: 20 days after silking; AF30: 30 days after silking; AS40: 40 days after silking; AS50: 50 days after silking; 2012B: Beijing in 2012; 2012H: Hainan in 2012; 2013B: Beijing in 2013; the 5-fold cross-validation scheme was implemented in this case

Prediction accuracies in the HO population using the UV model ranged from 0.06 to 0.52, and a minimal value was estimated using phenotypic data from 2012H. The *r*_MP_ estimated in stage V10 in three environments was lower than in other stages (Fig. 7a, Additional file 1: Table S8). As for the performance of the UV model in the LR population, *r*_MP_ changed from 0.38 to 0.58 in various stages across environments (Additional file 1: Table S8). However, improvement of *r*_MP_ could be achieved relative to its estimation by the UV model when the QTL detected in each stage within different environments were considered as fixed effects in the GBLUP model. The maximum difference of *r*_MP_ between the UV and FIXED models was 0.21 in stage V10 within 2012H. In general, the *r*_MP_ values based on the FIXED model were higher than those calculated by the UV model. Moreover, the changes of *r*_MP_ evaluated by the FIXED model ranged from 0.26 to 0.63 in the HO population and from 0.41 to 0.61 in the LR population (Fig. 7a, Additional file 1: Table S8). Compared to the FIXED model, the ME model could further enhance the prediction accuracy, for which the phenotypic data of RPR investigated in the first two environments were used to construct auxiliary variates in the multivariate model. The improvement of *r*_MP_ between the FIXED and ME models ranged from 0.02 to 0.17 in each stage (Fig. 7a). Furthermore, the proportions of residual variance estimated by the FIXED and ME models were lower than those calculated by the UV model, which ranged from 0.43 to 0.71 in each stage. However, the proportions of residual variance evaluated by the FIXED model were overall higher than those in the ME model (Additional file 1: Table S9). Additionally, cross-validation was performed by the MS model using the RPR values evaluated in the first six stages as auxiliary variates to predict RPR in the seventh stage. The *r*_MP_ was significantly increased in both RIL populations relative to its value estimated by the UV model (Fig. 7b). In addition, the proportions of variance components corresponding to the auxiliary variates in the MS model were very high, which can explain most of the phenotypic variance (Additional file 1: Table S10).

## Discussion

Stalk strength is a highly important agronomic trait in maize because of its relationship with stalk lodging and grain yield. However, RPR, as a crucial measurement index, can efficiently and precisely evaluate stalk strength to improve the lodging-resistance of breeding lines. Hence, the genetic dissection of RPR can provide powerful assistance for the selection of candidate lines with high stalk strength based on functional molecular marker detected by association and linkage mapping [1, 16, 20, 28, 29]. Furthermore, the utilization of genomic selection can also accelerate the breeding process of complex traits without phenotyping in later breeding phases [36–39]. Taking full advantage of genomic information led to better genomic prediction of RPR in this study.

The relatively higher stalk strength in 2012B compared to that in other environments is likely attributed to the lower planting density in that environment, indicating that a high planting density may reduce RPR, which is consistent with a previous study [6]. According to the ANOVA results, RPR has relatively high broad-sense heritability, which is supported by several previous studies [1, 19, 20, 26, 27], illustrating that genetic effects can account for the most proportion of phenotypic variance in RPR, and that better selection of RPR can be achieved in early generations if target lines are used as parents to construct breeding populations to screen out varieties with high stalk strength. However, RPR, a complex quantitative trait, is controlled by multiple genes with minor effects, which has been discussed in previous studies [1, 19, 28, 29]. The breeding scheme of population recurrent selection may be more efficient for the pyramid of favorable alleles related to RPR [8, 15, 25]. In brief, combining early generation selection and population improvement can enhance the breeding efficiency of selecting breeding lines with high stalk strength. But a lower broad-sense heritability was estimated in stage V10 in each RIL population, which may be attributed to the fact that stage V10 is a vegetative growth stage and nutrient and dry weights greatly increase in this stage [40]. Individual plants have weak stalk strength due to the rapid growth of the internodes, which can be affected by nutrient deficiencies, heat, and drought [41]. As illustrated by the ANOVA results in the present study, nongenetic effects account for a higher proportion of phenotypic variance of RPR in stage V10. According to the results of the phenotypic clustering and correlation analyses, stage V10 was individually separated from other stages and was associated with other stages with lower correlation coefficients, which further indicated that RPR in various stages may be controlled by different genetic factors and that the last six stages might have a similar genetic basis. Nevertheless, DTS was not classified into common subgroup with other stages after silking, which was likely attributed to the fact that the latter stages belonged to the phase of kernel development undergoing grain filling to maturity. On the other hand, the difference in RPR values between stages except for stage V10 was small, as shown by the distribution boxplot of each RIL population. Moreover, the broad-sense heritability in these stages was relatively higher than it was in stage V10. Hence, RPR measured in the silking phase or stage after silking can be used to evaluate stalk strength, as shown by several previous studies that had been provided evidence directly and were performed in silking phase or a few weeks after flowering [4, 20, 23, 26, 28, 29, 42]. Finally, inbred lines with high stalk strength in this study can be selected as novel germplasms to make candidate crosses in the future.

Genetic maps of each RIL population were constructed by the R package based on the Kosambi mapping function. Classical and cloned genes, including *P1* [31, 32] and *R1* [33, 34], were detected in each RIL population, indicating that these constructed linkage maps had high quality and accuracy to allow subsequent analysis of QTL mapping. The broad-sense heritability of RPR varied from stage to stage and was positively correlated with the number of QTL detected in each stage. It is implied that more QTL can be identified to better dissect the genetic basis of complex traits if a high broad-sense heritability is estimated for the target traits. On the other hand, more overlapped and common QTL for RPR can be obtained between different stages when the genetic correlation coefficient of both stages is increasingly large. In general, the higher the genetic correlation between traits, the more common the QTL, which may be illustrated by the fact that these traits were controlled by alike or linked genes or had common metabolic pathways [43]. The position and number of QTL detected in each experimental population were generally different across stages and environments. It is implied that discrepant genetic mechanisms may exist for RPR, which has been investigated in various situations, and it is further indicated that gene expression may be characterized by spatiotemporal specificity and is activated at specific times during plant development. Besides, the phenotypic variance explained by each detected QTL was lower than 15%, which was consistent with the results of other studies [1, 19], indicating that RPR is controlled by multiple alleles with minor effects and that there is a lack of major QTL for this trait. However, there were 18 pleiotropic QTL with overlapped genomic regions that were identified in multiple stages. In particular, pQTL6–1 in the HO population was repeatedly detected 16 times in different stages across environments. In addition, the pleiotropic QTL, namely, pQTL8, was identified 9 times across various phases and environments, including 8 times in the LR population and one time in the HO population. This phenomenon illustrates that certain alleles related to RPR are steadily expressed across stages during the development of maize and contribute to the formation of stalk strength throughout the entire growth period. From another perspective, several QTL detected in this study, including *qAhb1–2*, *qAhg2*, *qAhe2*, *qBhe2–1*, and *qBhc3*, were identified and consistent with previous studies in which discrepant populations and genotypic data were used to perform association or linkage mapping to explore the genetic architecture of RPR [20, 27–29], which provides further support for the topic mentioned above that some QTL associated with RPR are steadily expressed in diverse experimental populations. These loci in the genome may be regarded as candidate genomic regions and can likely be used to perform fine mapping and identify functional genes to dissect the genetic mechanism of RPR. Additionally, the relatively obvious difference in QTL mapping for RPR among the experimental populations was determined according to the results of this study and other previous studies [1, 19, 21, 26]. A reasonable explanation of this difference is as follows: first, RPR is regarded as a complex quantitative trait with an intricate genetic mechanism. There may be epistatic effects in which a QTL can interact with one QTL in this experimental population and with another locus in other genetic background, so that the QTL will produce different genetic effects in different populations; the second explanation is that the QTL related to target traits can be legitimately detected following segregation and recombination within this region. In other words, the associated QTL cannot be identified in a situation in which both parents of an experimental population have identical alleles at a QTL; the third explanation is that many QTL with minor genetic effects will not be detected repeatedly because they likely lack sufficient statistical power for QTL mapping [1, 44]. Hence, further research is needed to break RPR into a few direct components or sub-factors that can be used to more effectively dissect the genetic basis and explore candidate genes for stalk strength with the purpose of providing advice for marker-assisted breeding.

As an efficient approach for exploring the genetic architecture of target traits, linkage mapping has been widely applied to identify QTL and explore functional genes in molecular genetics research. The identified QTL can be used to develop molecular markers to assist the practical breeding and accelerate the selection process. Several primary candidate genes were found in the MaizeGDB database that corresponded to RPR in this study. One candidate gene within pQTL6–2 with the gene model ID GRMZM2G031200 is located on chromosome 6 with a physical position of 164.69 Mb. The homologs of this gene in *Arabidopsis* encode regulated transcription factors, namely, secondary wall-associated NAC domain protein1 (SND1), which is required for the normal biosynthesis of the secondary wall and is a critical transcriptional switch to activate this developmental program. The SND1 combines with other transcription factors to constitute a transcriptional network that regulates downstream targets that affect the biosynthesis of the secondary wall in fibers [45]. Moreover, two candidate genes were detected within the genomic region of pQTL6–1 consisting of seven RPR-related QTL with the model IDs GRMZM2G027723 and GRMZM2G135108 that are relevant to the formation of cell wall components. The first gene is *ZmCesA-2*, which is required to produce cellulose and is involved in primary wall biosynthesis [46, 47]. The another gene, namely *ZmPox3*, is a critical gene in the process of lignin biosynthesis and is involved in monolignol polymerization and exerts a positive effect on cell wall digestibility [48]. In addition, a candidate gene located in pQTL4–2, *ZmFBL41*, has the biological function of resistance to banded leaf and sheath blight and indirectly influences the accumulation of lignin. This gene encodes an F-box protein (ZmFBL41) that interacts with the protein ZmCAD, and its knockout has a negative effect on ZmCAD degradation and thus promotes lignin biosynthesis and restricts lesion expansion [49]. These descriptions indicate that candidate genes corresponding to cell wall components may regulate and determine the formation of RPR. On the other hand, several studies have reported the results of QTL mapping for cell wall components [26, 50–52], and some of these QTL have overlapped confidence intervals with the QTL identified in this study. Regarding pQTL6–2 detected in the HO population, its genomic region is consistent with the physical position of QTL associated with lignin, acid detergent fiber (ADF), neutral detergent fiber (NDF), acid detergent lignin/NDF, and in vitro dry matter digestibility (IVDMD) identified in previous studies [50, 51, 53–55]. Based on the results of related studies, pQTL4–2 has a physical region that overlaps with other loci that are associated with IVDMD and lignin [49, 51], and the interval of pQTL8 is consistent with the QTL related to IVDMD, which have negative relationships with lignin content [26, 52]. Hence, this evidence implies that certain QTL have pleiotropic effects and can control both RPR and the content of cell wall components, which likely indicates that RPR is closely associated with cell wall components, such as cellulose, hemicelluloses, and lignin, consistent with the results of previous studies [20, 26, 28]. In addition, the results of the GO and KEGG analyses provide further support for the abovementioned scenario because the enrichment items and metabolic pathways associated with cellar components and the formation of the cell wall were identified in this study. Consequently, candidate genes relevant to RPR are likely involved in the regulation and control of cell wall components, which may exert an important effect to improve RPR.

Genomic selection has been recognized as an efficient approach to select for complex traits in comparison with conventional marker-assisted selection [37, 38, 56, 57]. In the present study, the prediction accuracy estimated in each stage and population was obviously different when the UV model was used to perform cross-validation, which was likely attributed to the different estimates of broad-sense heritability in various situations. This phenomenon is in accordance with previous studies illustrating that broad-sense heritability is an important factor that impacts the evaluation of prediction accuracy [58–61]. The information on functional loci identified by linkage mapping can be used as fixed effects in the GS model to improve the predictive ability of models, which was performed in this study and in previous research [62–65]. However, the prediction accuracy was increased when the fixed effects model was implemented using the QTL that explained the proportion of phenotypic variance lower than 10%, which was consistent with a previous study [66]. This result illustrates that QTL related to target traits have the potential ability to improve prediction accuracy and should be assigned important roles in the models. A remarkable improvement of prediction accuracy was achieved in this study when the multivariate model was applied to perform the GS. Several previous studies have shown that using correlated traits as auxiliary variates in the GS model can efficiently enhance the prediction accuracy and is obviously superior to the univariate model [67–71]. The increase of prediction accuracy estimated by the FIXED and multivariate models is mainly attributed to the higher proportion of genetic variance captured by these models than in the univariate model, as shown in the present study and previous researches [67, 72]. Another explanation may be that multivariate models likely capture both additive and nonadditive interaction effects by using auxiliary covariates in the models [73]. In brief, information on genetic dissection or additional auxiliary variates can be integrated into improved models to enhance the selection efficiency of complex agronomic traits, such as yield, RPR and other resistance-relevant traits. Hence, several advices for GS-assisted breeding programs can be concluded to improve selection efficiency and further enhance the genetic gain per breeding cycle. Regarding the target traits with complex genetic architecture, the first point is that the information of functional markers developed based on cloned genes or validated QTL can be applied to modified models to improve the precision of the estimated marker effects; the second point is that the information from genetic correlated traits can be used to achieve a higher prediction accuracy for the target trait, namely, by using other traits as auxiliary variates in statistical models; and the last point is that historical data accumulated by breeding experiments can be used to capture the interaction effects between the environment and genotype for the purpose of increasing the predictive ability of GS models. These points may allow better selection of candidate lines with good performance in practical GS-assisted breeding schemes.

## Conclusions

Stalk lodging severely impacts plant standability and grain yield in maize. Stalk strength is an important agronomic trait and has a crucial effect on the improvement of lodging-resistance in modern maize breeding, and strong stalks can contribute to reduce lodging and achieve a harvestable yield. In the present study, phenotypic values evaluated in three environments and genotypic data identified by SNP array from two RIL populations were used to perform genetic dissection and genomic selection for rind penetrometer resistance. RPR has high genetic complexity and varies from stage to stage. Higher broad-sense heritability was obtained when RPR was investigated in the silking phase and stages after silking; these stages could be selected to better evaluate RPR for candidate lines. According to the QTL mapping results, RPR as a quantitative trait is controlled by multiple genes with minor effects. However, several QTL hotspots were identified in multiple stages across different environments, which might be able to be applied to develop functional markers to implement MAS for the selection of breeding lines. Furthermore, the annotation of candidate genes was based on the MaizeGDB database, and these candidates were usually involved in the regulation and formation of cell wall components. In addition, various models considering fixed effects or auxiliary variates were implemented to perform cross-validation, which achieved a remarkable improvement in prediction accuracy compared to the univariate model. Finally, the illustration of linkage mapping and genomic selection can provide pertinent suggestions for improving stalk strength and further enhancing lodging-resistance in maize breeding.

## Methods

### Plant materials

This experiment was performed using two RIL populations. The first was derived from crossing B73 and BY804, which consisted of 188 RILs. B73 is a famous elite line developed from the Iowa Stiff Stalk Synthetic population. BY804 is a special inbred line with a high kernel oil content, which was derived from a Beijing high-oil population. This RIL population was obtained from China Agricultural University. Another RIL population was composed of 215 lines, which were derived from a lodging-resistant maize hybrid with the elite inbred lines Zheng58 and HD568 as parents. Zheng58 is a famous inbred line that is the parent of the widely grown maize hybrid Zhengdan958. HD568 was purposefully selected with the criterion of high stalk strength. Finally, these two RIL populations are abbreviated as HO (high-oil) and LR (lodging-resistance) for simplicity.

### Field trial and phenotyping

A randomized incomplete block design with two replicates was implemented for the trial in each year. For the HO population, an experiment was primarily performed in Beijing in the summer of 2012, in which all lines were sown in a single row and the planting density was 49,500 plants per hectare. In addition, both RIL populations were evaluated in Hainan Province in the winter of 2012 and Beijing in the summer of 2013. Furthermore, each line was assigned to a single-row plot, and the planting density was 60,000 plants per hectare. The RPR in seven stages, including the tenth-leaf stage (V10), days to silking (DTS), and 10 to 50 days after silking (AS10, AS20, AS30, AS40 and AS50), was evaluated in the middle of the flat side of the third stalk internode aboveground with an electronic rind penetrometer (AWOS-SL04, Aiwoshi Science & Technology Co., Ltd. Company, Hebei, China). In this experiment, two to five randomly selected plants were used to investigate RPR measurements, and the RPR of each line was determined by the mean of ten measures.

### Phenotypic data analysis

**Analysis of variance, broad-sense heritability and best linear unbiased estimation.**

Analysis of variance (ANOVA) was performed using the *aov* function in the R package stats version 3.6.0 (R Core Team, 2019). The linear model is as follows:

$$Y_{ijk} = \mu + g_{i} + e_{j} + ge_{ij} + r_{k(j)} + \varepsilon_{ijk},$$

where *y*_*ijk*_ is the phenotypic values of target trait, μ is the grand mean, *g*_*i*_ is the genetic effect of the *i*^*th*^ genotype, *e*_*j*_ is the environmental effect of the *j*^*th*^ environment, *ge*_*ij*_ is the interaction effect between the *i*^*th*^ genotype and the *j*^*th*^ environment, *r*_*jk*_ is the effect of the *k*^*th*^ replicate, and *ε*_*ijk*_ is the residual error. Broad-sense heritability was estimated according to the formula:

$$H^{2} = \sigma^{2}_{\text{g}}/\left(\sigma^{2}_{\text{g}}+\sigma^{2}_{\text{ge}}/e + \sigma^{2}_{\varepsilon}/re \right), $$

where ${\sigma}_{\mathrm{g}}^2$, ${\sigma}_{ge}^2$, and ${\sigma}_{\varepsilon}^2$ are the variance components of genotype, genotype by environment interaction and random error, respectively, and *r* and *e* are the numbers of replicates and environments, respectively. In addition, the R package *lme4* version 1.1–21 was used to perform the best unbiased linear estimation (BLUE) for the genetic effects with the following mixed linear model (MLM) [74, 75]:

$$ {y}_{ij k}=\upmu +{g}_i+{e}_j+g{e}_{ij}+{r}_{jk}+{\varepsilon}_{ij k}, $$

which all components were described based on Liu et al. (2019) [76]. The phenotypic values of RPR determined in the winter of 2012 and the summer of 2013 were jointly used to perform ANOVA and estimate the BLUE values. The BLUE values were used to perform QTL mapping to detect the relationship between *H*^*2*^ and the QTL number.

### Construction of the hierarchical clustering of RPR in various stages

For the construction of the hierarchical clustering of RPR in two RIL populations, the BLUE values of each stage were first standardized to a zero mean and unit variance according to the following formula:

$$ {Y}_{ij}=\left({X}_{ij}- mean\left({X}_{.j}\right)\right)/ sd\left({X}_{.j}\right), $$

where *Y*_*ij*_ is the transformed value, *X*_*ij*_ is the BLUE values of the *i*^*th*^ genotype in the *j*^*th*^ stage, mean() is defined as the mean value, and sd() is the standard deviation. Furthermore, the transformed values of RPR in each stage were used to calculate the Euclidean distances between all pairs of stages, which was performed by the euclidean method with the *dist* function in the R package stats version 3.6.0. The formula is as follows [43]:

$$ {D}_{AB}={\left({\sum}_{i=1}^{\mathrm{n}}{\left({Y}_{iA}-{Y}_{iB}\right)}^2\right)}^{1/2}, $$

where *D*_*AB*_ is the value of the Euclidean distance between stages A and B; *Y*_*iA*_ and *Y*_*iB*_ are the transformed values of the *i*^*th*^ genotype in stages A and B, respectively; and n is the individual numbers of two RIL populations. The *hclust* function was used to construct the hierarchical clustering tree based on the distance values of all pairs of seven stages.

### Phenotypic and genetic correlation

The phenotypic correlation (*r*_*p*_) is a measurement of the association between the phenotypic values of individuals for a pair of targeted traits. The genetic correlation (*r*_*g*_) is a parameter of genetics that can be used to evaluate the degree of association in genetic variation between two traits. The correlation coefficients are estimated by the following formulae [77–79]:

$$ {r}_p= co{v}_p\left(A,B\right)/\sqrt{v_{pA}{v}_{pB}}; $$

$$ {r}_g= co{v}_g\left(A,B\right)/\sqrt{v_{gA}{v}_{gB}}; $$

where cov_*p*_() and cov_*g*_() are the phenotypic and genetic covariances between traits, respectively, and *V*_*p*_ and *V*_*g*_ are the phenotypic and genetic variances of target traits, respectively. Phenotypic and genetic correlation analyses were performed using the *cor* function in the R package *stats* version 3.6.0 (R Core Team, 2019) and the *asreml* function in the R package *ASReml* version 3.0 [80], respectively. Phenotypic data of RPR evaluated in each environment were utilized to estimate the phenotypic correlation coefficient. However, the RPR values determined in the winter of 2012 and the summer of 2013 were used to perform an analysis of genetic correlation.

### Genotypic data analysis

#### Genotyping and quality control

For the HO population, all inbred lines were used for genotyping with the MaizeSNP3K array, which is a subset of the Illumina MaizeSNP50 BeadChip [81]. Markers with missing rates greater than 0.20 and minor allele frequencies (MAFs) less than 0.05 were removed. However, the maize 55 K SNP array [82] was used to genotype all the RILs in the LR population. The process of quality control for genotypic data in LR population was based on Liu et al. (2019) [76]. Moreover, chi-square tests were performed for all the SNPs in each population with the aim of filtering out markers with segregation distortion (*P* < 0.05) in the two RIL populations.

### Construction of the bin map and QTL mapping

Bin markers were detected and aligned with the sliding-window approach, which was applied to identify variant calling errors and evaluate the ratio of SNP alleles derived from the parents. The detailed method of bin map construction was described as Liu et al. (2019) [76], which was based on several studies [83, 84]. The genetic maps of both RIL populations were constructed by the Kosambi mapping function in the *mstmap* function in the R package *ASMap* version 1.0–4 [85]. QTL related to RPR were detected by composite interval mapping using the *cim* function in the R package *R/qtl* version 1.44–9 [35]. The threshold of logarithm of the odds (LOD), confidence interval of each QTL, and pleiotropic QTL were analyzed and determined according to several studies [76, 83]. Furthermore, the most likely candidate genes within the confidence interval were consulted and selected from the maize genetics and genomics database (MaizeGDB, https://www.maizegdb.org/). Because fewer qualified markers were retained in the HO population, these SNPs were directly used to construct a linkage map and perform further analyses without detecting bin markers. However, the bin markers could be checked and used to perform subsequent analyses in the LR population.

### Analysis of GO enrichment and KEGG pathway

Gene ontology (GO) enrichment analysis was performed using singular enrichment analysis (SEA) by AgriGO version 2.0 (http://systemsbiology.cau.edu.cn/agriGOv2/ index.php) with the Fisher statistical test method and Yekutieli multitest adjustment method at a significance level of *P* < 0.05 [86]. Additionally, the slim of GO function was summarized by GOSlimViewer (https://agbase.arizona.edu/cgi-bin/tools/goslimviewer_select.pl) of AgBase [87]. For Kyoto Encyclopedia of Genes and Genomes (KEGG) pathway annotation, KOBAS version 3.0 (http://kobas.cbi.pku.edu.cn/index.php) was used to perform an analysis of the functional gene set enrichment with Fisher statistical method and the Benjamini and Yekutieli FDR (false discovery rate) correction method (*P* < 0.05). Significant GO items and KEGG entries were extracted to draw plots.

### Genomic selection

A 5-fold cross-validation scheme with 100 replicates was used to assess the performance of each model and calculate the prediction accuracy (*r*_MP_), which was the correlation coefficient between genomic-estimated breeding values (GEBVs) and phenotypic values. Three models were used to perform cross-validation, which were developed from the genomic best linear unbiased prediction (GBLUP) model. The univariate model (UV) is essentially a general form of the GBLUP model, and the mixed model is as follows [88, 89]:

$$y = 1_{n}\mu + u + \varepsilon ,$$

where ***y*** is a vector (*n* × 1) of phenotypic values, 1_*n*_ is a vector (*n* × 1) of ones, μ is the overall mean, ***u*** is the random effects that obeys a normal distribution *N*(0,***G***${\sigma}_u^2$), ${\sigma}_u^2$ is the genetic variance, ***G*** is the genomic relationship matrix among all genotypes calculated following VanRaden (2008) [88], *ε* is a vector (*n* × 1) of random terms with a normal distribution *N*(0,***I***${\sigma}_{\varepsilon}^2$), and ***I*** is an identity matrix. In addition, *n* is the number of individuals. For the GBLUP model including fixed effects (FIXED), the formula can be described as [76]:

$$ \boldsymbol{y}=\boldsymbol{X}\boldsymbol{\beta } +\boldsymbol{u}+\varepsilon, $$

where ***β*** is the vector (*n* × 1) of fixed effects, ***X*** is the (*n* × *p*) design matrix, and the other parameters are identical to the description mentioned above. All markers with peak LOD value of QTL in each target trait were selected to construct the ***X*** design matrix, and *p* denotes the number of target markers. However, the ***G*** matrix is calculated by the design matrix containing *m*-*p* markers, where *m* is the number of all markers in each RIL population. Finally, the multivariate model was developed from the univariate model, and the model was as follows [67]:

$$y = 1_{n}\mu + u + v + \varepsilon ,$$

where ***y*** is a vector (*n* × 1) of the target variate, ***v*** is the random effects for auxiliary variates with a normal distribution *N*(0,***G***_***v***_${\sigma}_v^2$), ${\sigma}_v^2$ is the variance component of ***v***, and the other parameters are identical to the description mentioned above. The ***G***_***v***_ is the multivariate relationship matrix, which was calculated as follows: ***G***_***v***_ = n***M***_***v***_***M***_***v***_’/trace(***M***_***v***_***M***_***v***_’), where ***M***_***v***_ = [***y***_***1***_,***y***_***2***_,…,***y***_***i***_,...,***y***_***t-1***_], ***y***_***i***_ is a scaled vector (*n* × 1) of the phenotypic values of the *i*^th^ environment or stage that were standardized to zero mean and unit variance, *t* is the number of all variates, and trace denotes the sum of all diagonal elements. The phenotypic data of the t-1 environments or stages are recognized as auxiliary variates in the model. If auxiliary variates were derived from multiple environments, the multivariate model would be abbreviated as ME. For auxiliary variates derived from multiple stages, the model was represented as MS for short. These models were fitted using the R package *BGLR* version 1.0.8 [90], and the iteration of the Gibbs sampler was set to 10,000, with the first 5000 samples discarded as burn in.

## Supplementary information

**Additional file 1 : Table S1.** Analysis of variance (ANOVA) and broad-sense heritability for rind penetrometer resistance of various stages across environments in two RIL populations. **Table S2.** Summary of the bin map of LR population (Zheng58 × HD568). **Table S3.** Summary of the bins that were greater than 10.0 Mb in length in LR population (Zheng58 × HD568). **Table S4.** Summary of the high-density genetic map derived from two RIL populations. **Table S5.** QTL for rind penetrometer resistance in high-oil population (B73 × BY804). **Table S6.** QTL for rind penetrometer resistance in lodging-resistance population (Zheng58 × HD568). **Table S7.** Candidate genes annotation. **Table S8.** Comparison of prediction accuracies between models. **Table S9.** Proportion of variance components estimated by UV, FIXED and ME models. **Table S10.** Proportion of variance components estimated by UV and MS models. **Figure S1.** Phenotypic correlation of rind penetrometer resistance between all pairs of stages within each environment in two RIL populations. (A) to (C) High-oil population (B73 × BY804) in Beijing in 2012, Hainan in 2012 and Beijing in 2013. (D) to (E) Lodging-resistance population (Zheng58 × HD568) in Hainan in 2012 and Beijing in 2013. V10: the tenth-leaf stage; DTS: days to silking; AS10: 10 days after silking; AS20: 20 days after silking; AF30: 30 days after silking; AS40: 40 days after silking; AS50: 50 days after silking. **Figure S2.** Comparison of the physical map and genetic map constructed with bin markers in the high-oil population (B73 × BY804). The x-axis refers to the linear order of bins based on physical positions in the maize reference genome, and the y-axis denotes the order of bins based on genetic distance in the linkage map; LG: linkage group; Chr.: chromosome. **Figure S3.** Illustration of pQTL8 identified in various situations. Violin plots denote the difference between genotypes derived from each parent; HO: the high-oil population (B73 × BY804); LR: the lodging-resistance population (Zheng58 × HD568); RPR: rind penetrometer resistance; V10: the tenth-leaf stage; DTS: days to silking; AS10: 10 days after silking; AS20: 20 days after silking; AF30: 30 days after silking; AS40: 40 days after silking; AS50: 50 days after silking; 2012H: Hainan in 2012; 2013B: Beijing in 2013.

## Data Availability

Data supporting the results can be found in Additional file 1 and any other datasets used and/or analyzed during the current study is available from the corresponding author on reasonable request.

## Abbreviations

ANOVA: Analysis of variance
AS10 to AS50: 10 to 50 days after silking
BLUE: Best linear unbiased estimation
DTS: Days to silking
GO: Gene ontology
GS: Genomic selection
GWAS: Genome-wide association study
HO: High-oil
KEGG: Kyoto encyclopedia of genes and genomes
LOD: Logarithm of the odds
LR: Lodging-resistance
MAF: Minor allele frequency
MAS: Marker-assisted selection
QTL: Quantitative trait loci
RIL: Recombinant inbred line
RPR: Rind penetrometer resistance
SNP: Single nucleotide polymorphism
SSR: Simple sequence repeats
V10: Tenth-leaf stage

## References

[1] Flint-Garcia SA, Jampatong C, Darrah LL, McMullen MD (2003). Quantitative trait locus analysis of stalk strength in four maize populations. Crop Sci.

[2] Ennos AR, Crook MJ, Grimshaw C (1993). The anchorage mechanics of maize, *Zea mays*. J Exp Bot.

[3] Esechie HA, Rodriguez V, Al-Asmi H (2004). Comparison of local and exotic maize varieties for stalk lodging components in a desert climate. Eur J Agron.

[4] Ma D, Xie R, Liu X, Niu X, Hou P, Wang K (2014). Lodging-related stalk characteristics of maize varieties in China since the 1950s. Crop Sci.

[5] Mihm JA (1985). Breeding for host plant resistance to maize stem-borers. Int J Trop Insect Sci.

[6] Xue J, Zhao Y, Gou L, Shi Z, Yao M, Zhang W (2016). How high plant density of maize affects basal internode development and strength formation. Crop Sci.

[7] Colbert TR, Darrah LL, Zuber MS (1984). Effect of recurrent selection for stalk crushing strength on agronomic characteristics and soluble stalk solids in maize. Crop Sci.

[8] Dudley JW (1994). Selection for rind puncture resistance in two maize populations. Crop Sci.

[9] Jampatong S, Darrah LL, Krause GF, Barry BD (2000). Effect of one- and two-eared selection on stalk strength and other characters in maize. Crop Sci.

[10] Kamran M, Cui W, Ahmad I, Meng X, Zhang X, Su W (2018). Effect of paclobutrazol, a potential growth regulator on stalk mechanical strength, lignin accumulation and its relation with lodging resistance of maize. Plant Growth Regul.

[11] Kang MS, Din AK, Zhang Y, Magari R (1999). Combining ability for rind puncture resistance in maize. Crop Sci.

[12] Gou L, Huang J, Zhang B, Li T, Sun R, Zhao M (2007). Effects of population density on stalk lodging resistant mechanism and agronomic characteristics of maize. Acta Agron Sin.

[13] Thompson DL (1963). Stalk strength of corn as measured by crushing strength and rind thickness. Crop Sci.

[14] Sibale EM, Darrah LL, Zuber MS (1992). Comparison of two rind penetrometers for measurement of stalk strength in maize. Maydica..

[15] Martin SA, Darrah LL, Hibbard BE (2004). Divergent selection for rind penetrometer resistance and its effects on European corn borer damage and stalk traits in corn. Crop Sci.

[16] Hu H, Liu W, Fu Z, Homann L, Technow F, Wang H (2013). QTL mapping of stalk bending strength in a recombinant inbred line maize population. Theor Appl Genet.

[17] Zuber MS, Grogan CO (1961). A new technique for measuring stalk strength in corn. Crop Sci.

[18] Ma Q-H (2009). The expression of caffeic acid 3-O-methyltransferase in two wheat genotypes differing in lodging resistance. J Exp Bot.

[19] Hu H, Meng Y, Wang H, Liu H, Chen S (2012). Identifying quantitative trait loci and determining closely related stalk traits for rind penetrometer resistance in a high-oil maize population. Theor Appl Genet.

[20] Li K, Yan J, Li J, Yang X (2014). Genetic architecture of rind penetrometer resistance in two maize recombinant inbred line populations. BMC Plant Biol.

[21] Flint-Garcia SA, McMullen MD, Darrah LL (2003). Genetic relationship of stalk strength and ear height in maize. Crop Sci.

[22] Gou L, Huang J, Sun R, Ding Z, Dong Z, Zhao M (2010). Variation characteristic of stalk penetration strength of maize with different density-tolerance varieties. Trans Chin Soc Agric Eng.

[23] Flint-Garcia SA, Darrah LL, McMullen MD, Hibbard BE (2003). Phenotypic versus marker-assisted selection for stalk strength and second-generation European corn borer resistance in maize. Theor Appl Genet.

[24] Butrón A, Malvar RA, Revilla P, Soengas P, Ordás A, Geiger HH (2002). Rind puncture resistance in maize: inheritance and relationship with resistance to pink stem borer attack. Plant Breed.

[25] Albrecht B, Dudley JW (1987). Divergent selection for stalk quality and grain yield in an adapted × exotic maize population cross. Crop Sci.

[26] Meng Y, Li J, Liu J, Hu H, Li W, Liu W (2016). Ploidy effect and genetic architecture exploration of stalk traits using DH and its corresponding haploid populations in maize. BMC Plant Biol.

[27] Zhang Y, Liang T, Chen M, Zhang Y, Wang T, Lin H (2019). Genetic dissection of stalk lodging-related traits using an IBM Syn10 DH population in maize across three environments (*Zea mays* L.). Mol Gen Genomics.

[28] Peiffer JA, Flint-Garcia SA, Leon ND, McMullen MD, Kaeppler SM, Buckler ES (2013). The genetic architecture of maize stalk strength. PLoS One.

[29] Zhang Y, Liu P, Zhang X, Zheng Q, Chen M, Ge F (2018). Multi-locus genome-wide association study reveals the genetic architecture of stalk lodging resistance-related traits in maize. Front Plant Sci.

[30] Feng G, Liu Z, Wu Y, Li Y, Huang C (2010). Primary study on correlation between corn variety lodging resistances and its stem puncture-pull strength. J Maize Sci.

[31] Grotewold E, Athma P, Peterson T (1991). A possible hot spot for *Ac* insertion in the maize *P* gene. Mol Gen Genet.

[32] Zhang F, Peterson T (2005). Comparisons of maize *pericarp color1* alleles reveal paralogous gene recombination and an organ-specific enhancer region. Plant Cell.

[33] Scanlon MJ, Stinard PS, James MG, Myers AM, Robertson DS (1994). Genetic analysis of 63 mutations affecting maize kernel development isolated from Mutator stocks. Genetics..

[34] Walker EL (1998). Paramutation of the *r1* locus of maize is associated with increased cytosine methylation. Genetics..

[35] Arends D, Prins P, Jansen RC, Broman KW (2010). R/QTL: high-throughput multiple QTL mapping. Bioinformatics..

[36] Meuwissen THE, Hayes BJ, Goddard ME (2001). Prediction of total genetic value using genome-wide dense marker maps. Genetics..

[37] Crossa J, Pérez-Rodríguez P, Cuevas J, Montesinos-López O, Jarquín D (2017). de los Campos G, et al. genomic selection in plant breeding: methods, models, and perspectives. Trends Plant Sci.

[38] Desta ZA, Ortiz R (2014). Genomic selection: genome-wide prediction in plant improvement. Trends Plant Sci.

[39] Jonas E, de Koning D-J (2013). Does genomic selection have a future in plant breeding?. Trends Biotechnol.

[40] Hume DJ, Campbell DK (1972). Accumulation and translocation of soluble solids in corn stalks. Can J Plant Sci.

[41] Hanway JJ (1963). Growth stages of corn (*Zea mays* L.). Agron J.

[42] Xue J, Gou L, Shi Z, Zhao Y, Zhang W (2017). Effect of leaf removal on photosynthetically active radiation distribution in maize canopy and stalk strength. J Integr Agric.

[43] Pan Q, Xu Y, Li K, Peng Y, Zhan W, Li W (2017). The genetic basis of plant architecture in 10 maize recombinant inbred line populations. Plant Physiol.

[44] Beavis WD, Paterson AH (1998). QTL analyses: power, precision, and accuracy. Molecular dissection of complex traits.

[45] Zhong R, Lee C, Zhou J, McCarthy RL, Ye Z-H (2008). A battery of transcription factors involved in the regulation of secondary cell wall biosynthesis in Arabidopsis. Plant Cell.

[46] Persson S, Paredez A, Carroll A, Palsdottir H, Doblin M, Poindexter P (2007). Genetic evidence for three unique components in primary cell-wall cellulose synthase complexes in *Arabidopsis*. Proc Natl Acad Sci.

[47] Holland N, Holland D, Helentjaris T, Dhugga KS, Xoconostle-Cazares B, Delmer DP (2000). A comparative analysis of the plant cellulose synthase (*CesA*) gene family. Plant Physiol.

[48] Guillet-Claude C, Birolleau-Touchard C, Manicacci D, Rogowsky PM, Rigau J, Murigneux A (2004). Nucleotide diversity of the *ZmPox3* maize peroxidase gene: relationships between a MITE insertion in exon 2 and variation in forage maize digestibility. BMC Genet.

[49] Li N, Lin B, Wang H, Li X, Yang F, Ding X (2019). Natural variation in *ZmFBL41* confers banded leaf and sheath blight resistance in maize. Nat Genet.

[50] Wang Q, Li K, Hu X, Shi H, Liu Z, Wu Y, et al. Genetic analysis and QTL mapping of stalk cell wall components and digestibility in maize recombinant inbred lines from B73×By804. Crop J. 2019; 10.1016/j.cj.2019.06.009.

[51] Wang H, Li K, Hu X, Liu Z, Wu Y, Huang C (2016). Genome-wide association analysis of forage quality in maize mature stalk. BMC Plant Biol.

[52] Barrière Y, Méchin V, Lefevre B, Maltese S (2012). QTLs for agronomic and cell wall traits in a maize RIL progeny derived from a cross between an old Minnesota13 line and a modern Iodent line. Theor Appl Genet.

[53] Courtial A, Jourda C, Arribat S, Huguet S, Reymond M, Grima-Pettenati J (2012). Comparative expression of cell wall related genes in four maize RILs and one parental line of variable lignin content and cell wall degradability. Maydica..

[54] Courtial A, Méchin V, Reymond M, Grima-Pettenati J, Barrière Y (2014). Colocalizations between several QTLs for cell wall degradability and composition in the F288 × F271 early maize RIL progeny paise the question of the nature of the possible underlying determinants and breeding targets for biofuel capacity. BioEnergy Res.

[55] Li K, Wang H, Hu X, Liu Z, Wu Y, Huang C (2016). Genome-wide association study reveals the genetic basis of stalk cell wall components in maize. PLoS One.

[56] Voss-Fels KP, Cooper M, Hayes BJ (2019). Accelerating crop genetic gains with genomic selection. Theor Appl Genet.

[57] Cerrudo D, Cao S, Yuan Y, Martinez C, Suarez EA, Babu R (2018). Genomic selection outperforms marker assisted selection for grain yield and physiological traits in a maize doubled haploid population across water treatments. Front Plant Sci.

[58] Zhang A, Wang H, Beyene Y, Semagn K, Liu Y, Cao S (2017). Effect of trait heritability, training population size and marker density on genomic prediction accuracy estimation in 22 bi-parental tropical maize populations. Front Plant Sci.

[59] Combs E, Bernardo R (2013). Accuracy of genomewide selection for different traits with constant population size, heritability, and number of markers. Plant Genome.

[60] Liu X, Wang H, Wang H, Guo Z, Xu X, Liu J (2018). Factors affecting genomic selection revealed by empirical evidence in maize. Crop J.

[61] Heffner EL, Jannink J-L, Sorrells ME (2011). Genomic selection accuracy using multifamily prediction models in a wheat breeding program. Plant Genome.

[62] Bernardo R (2014). Genomewide selection when major genes are known. Crop Sci.

[63] Boeven PHG, Longin CFH, Leiser WL, Kollers S, Ebmeyer E, Würschum T (2016). Genetic architecture of male floral traits required for hybrid wheat breeding. Theor Appl Genet.

[64] Spindel JE, Begum H, Akdemir D, Collard B, Redoña E, Jannink J-L (2016). Genome-wide prediction models that incorporate de novo GWAS are a powerful new tool for tropical rice improvement. Heredity..

[65] Sarinelli JM, Murphy JP, Tyagi P, Holland JB, Johnson JW, Mergoum M (2019). Training population selection and use of fixed effects to optimize genomic predictions in a historical USA winter wheat panel. Theor Appl Genet.

[66] Arruda MP, Lipka AE, Brown PJ, Krill AM, Thurber C, Brown-Guedira G (2016). Comparing genomic selection and marker-assisted selection for Fusarium head blight resistance in wheat (*Triticum aestivum* L.). Mol Breed.

[67] Wang X, Li L, Yang Z, Zheng X, Yu S, Xu C (2017). Predicting rice hybrid performance using univariate and multivariate GBLUP models based on North Carolina mating design II. Heredity..

[68] Calus MP, Veerkamp RF (2011). Accuracy of multi-trait genomic selection using different methods. Genet Sel Evol.

[69] Jia Y, Jannink J-L (2012). Multiple-trait genomic selection methods increase genetic value prediction accuracy. Genetics..

[70] Guo G, Zhao F, Wang Y, Zhang Y, Du L, Su G (2014). Comparison of single-trait and multiple-trait genomic prediction models. BMC Genet.

[71] Engle BN, Corbet NJ, Allen JM, Laing AR, Fordyce G, McGowan MR (2019). Multivariate genomic predictions for age at puberty in tropically adapted beef heifers. J Anim Sci.

[72] Lehermeier C, Schön C-C, de los Campos G (2015). Assessment of genetic heterogeneity in structured plant populations using multivariate whole-genome regression models. Genetics.

[73] Okeke UG, Akdemir D, Rabbi I, Kulakow P, Jannink J-L (2017). Accuracies of univariate and multivariate genomic prediction models in African cassava. Genet Sel Evol.

[74] Yang J, Mezmouk S, Baumgarten A, Buckler ES, Guill KE, McMullen MD (2017). Incomplete dominance of deleterious alleles contributes substantially to trait variation and heterosis in maize. PLoS Genet.

[75] Bates D, Mächler M, Bolker B, Walker S (2015). Fitting linear mixed-effects models using lme4. J Stat Softw.

[76] Liu X, Wang H, Hu X, Li K, Liu Z, Wu Y (2019). Improving genomic selection with quantitative trait loci and nonadditive effects revealed by empirical evidence in maize. Front Plant Sci.

[77] Searle SR (1961). Phenotypic, genetic and environmental correlations. Biometrics..

[78] Hill WG, Maloy S, Hughes K (2013). Genetic correlation. Brenner’s encyclopedia of genetics.

[79] Hazel LN (1943). The genetic basis for constructing selection indexes. Genetics..

[80] Butler DG, Cullis BR, Gilmour AR, Thompson R. ASReml-R reference manual (version 3). Brisb State Qld Dep Prim Ind Fish. 2009.

[81] Ganal MW, Durstewitz G, Polley A, Bérard A, Buckler ES, Charcosset A (2011). A large maize (*Zea mays* L.) SNP genotyping array: development and germplasm genotyping, and genetic mapping to compare with the B73 reference genome. PLoS One.

[82] Xu C, Ren Y, Jian Y, Guo Z, Zhang Y, Xie C (2017). Development of a maize 55 K SNP array with improved genome coverage for molecular breeding. Mol Breed.

[83] Zhou Z, Zhang C, Zhou Y, Hao Z, Wang Z, Zeng X (2016). Genetic dissection of maize plant architecture with an ultra-high density bin map based on recombinant inbred lines. BMC Genomics.

[84] Huang X, Feng Q, Qian Q, Zhao Q, Wang L, Wang A (2009). High-throughput genotyping by whole-genome resequencing. Genome Res.

[85] Taylor J, Butler D (2017). R package ASMap: efficient genetic linkage map construction and diagnosis. J Stat Softw.

[86] Tian T, Liu Y, Yan H, You Q, Yi X, Du Z (2017). agriGO v2.0: a GO analysis toolkit for the agricultural community, 2017 update. Nucleic Acids Res.

[87] McCarthy FM, Wang N, Magee GB, Nanduri B, Lawrence ML, Camon EB (2006). AgBase: a functional genomics resource for agriculture. BMC Genomics.

[88] VanRaden PM (2008). Efficient methods to compute genomic predictions. J Dairy Sci.

[89] Bernardo R (1996). Best linear unbiased prediction of maize single-cross performance. Crop Sci.

[90] Pérez P, de los Campos G (2014). Genome-wide regression & prediction with the BGLR statistical package. Genetics..

