# Supplementary material for: Genetic mapping and genomic selection for maize stalk strength
Source: BMC Plant Biol. 2020 May 7;20:196. doi: 10.1186/s12870-020-2270-4 (PMC7204062; doi:10.1186/s12870-020-2270-4)
Supplement: Supplementary file 1 — Additional file 1 : Table S1. Analysis of variance (ANOVA) and broad-sense heritability for rind penetrometer resistance of various stages across environments in two RIL populations. Table S2. Summary of the bin map of LR population (Zheng58 × HD568). Table S3. Summary of the bins that were greater than 10.0 Mb in length in LR population (Zheng58 × HD568). Table S4. Summary of the high-density genetic map derived from two RIL populations. Table S5. QTL for rind penetrometer resistance in high-oil population (B73 × BY804). Table S6. QTL for rind penetrometer resistance in lodging-resistance population (Zheng58 × HD568). Table S7. Candidate genes annotation. Table S8. Comparison of prediction accuracies between models. Table S9. Proportion of variance components estimated by UV, FIXED and ME models. Table S10. Proportion of variance components estimated by UV and MS models. Figure S1. Phenotypic correlation of rind penetrometer resistance between all pairs of stages within each environment in two RIL populations. (A) to (C) High-oil population (B73 × BY804) in Beijing in 2012, Hainan in 2012 and Beijing in 2013. (D) to (E) Lodging-resistance population (Zheng58 × HD568) in Hainan in 2012 and Beijing in 2013. V10: the tenth-leaf stage; DTS: days to silking; AS10: 10 days after silking; AS20: 20 days after silking; AF30: 30 days after silking; AS40: 40 days after silking; AS50: 50 days after silking. Figure S2. Comparison of the physical map and genetic map constructed with bin markers in the high-oil population (B73 × BY804). The x-axis refers to the linear order of bins based on physical positions in the maize reference genome, and the y-axis denotes the order of bins based on genetic distance in the linkage map; LG: linkage group; Chr.: chromosome. Figure S3. Illustration of pQTL8 identified in various situations. Violin plots denote the difference between genotypes derived from each parent; HO: the high-oil population (B73 × BY804); LR: the lodging-resistance popula [file 12870_2020_2270_MOESM1_ESM.docx]

**Table S1** Analysis of variance (ANOVA) and broad-sense heritability for rind penetrometer resistance of various stages across environments in two RIL populations

| Pop.^a^ | Mean squares | | | | | | *H*^2^(%) |
| --- | --- | --- | --- | --- | --- | --- | --- |
|  | Stage^b^ | Env. | Rep. | Genotype | Geno.×Env. | Residual |  |
| HO | V10 | 135.68** | 0.02 | 0.14** | 0.08** | 0.06 | 51.8 |
|  | DTS | 0.73* | 1.20** | 0.91** | 0.31** | 0.16 | 70.5 |
|  | AS10 | 4.97** | 1.78** | 1.52** | 0.64** | 0.22 | 67.0 |
|  | AS20 | 1.07* | <0.01 | 1.69** | 0.50** | 0.23 | 74.5 |
|  | AS30 | 2.03** | 0.06 | 1.40** | 0.49** | 0.22 | 70.7 |
|  | AS40 | 1.11* | <0.01 | 1.21** | 0.49** | 0.19 | 67.5 |
|  | AS50 | 9.20** | 1.96** | 1.19** | 0.47** | 0.22 | 66.9 |
| LR | V10 | 220.07** | 0.12 | 0.21** | 0.12** | 0.06 | 56.4 |
|  | DTS | 33.98** | 0.45 | 0.74** | 0.26** | 0.14 | 70.2 |
|  | AS10 | 42.40** | 0.64 | 1.21** | 0.41** | 0.22 | 70.7 |
|  | AS20 | 11.48** | 0.13 | 1.08** | 0.42** | 0.19 | 68.3 |
|  | AS30 | 40.08** | <0.01 | 0.96** | 0.33** | 0.17 | 70.8 |
|  | AS40 | 5.16** | 0.31 | 0.75** | 0.26** | 0.13 | 70.9 |
|  | AS50 | 20.17** | 0.15 | 0.83** | 0.33** | 0.15 | 67.1 |

^a^ Pop.: experimental populations. HO: the high-oil population (B73×BY804); LR: the lodging-resistance population (Zheng58×HD568).

^b^ V10: the tenth-leaf stage; DTS: days to silking; AS10: ten days after silking; AS20: twenty days after silking; AF30: thirty days after silking; AS40: forty days after silking; AS50: fifty days after silking. Env.: environments. Rep: replicates. Geno.: genotype. **: *P* < 0.01. *H*^2^: broad-sense heritability.

**Table S2** Summary of the bin map of LR population (Zheng58×HD568)

| Chr.^a^ | No. of bin markers^b^ | Physical length of map (Mb) | Average length of bin markers (Mb) | Minimal length of bin markers (Kb) | Maximal length of bin markers (Mb) |
| --- | --- | --- | --- | --- | --- |
| 1 | 412 | 301.1 | 0.73 | 5.7 | 19.8 |
| 2 | 261 | 237.0 | 0.91 | 5.8 | 11.6 |
| 3 | 286 | 232.1 | 0.81 | 5.4 | 23.5 |
| 4 | 202 | 246.9 | 1.22 | 6.2 | 23.6 |
| 5 | 244 | 217.7 | 0.89 | 5.2 | 29.9 |
| 6 | 158 | 169.2 | 1.07 | 5.2 | 16.5 |
| 7 | 183 | 176.1 | 0.96 | 5.1 | 52.6 |
| 8 | 184 | 175.6 | 0.95 | 6.7 | 40.7 |
| 9 | 99 | 155.3 | 1.56 | 8.2 | 11.1 |
| 10 | 92 | 149.9 | 1.62 | 6.6 | 54.4 |
| Overall | 2121 | 2060.9 | 0.97 | 5.1 | 54.4 |

^a^ Chr.: the number of chromosomes.

^b^ No. of bins: the number of bin markers on the chromosome.

**Table S3** Summary of the bins that were greater than 10.0 Mb in length in LR population (Zheng58×HD568)

| Marker name | Chr.^a^ | Physical position (Mb) | Bin length (Mb) | Description | Centromeric region (B73 RefGen_V3, Mb) |
| --- | --- | --- | --- | --- | --- |
| lmk0200 | 1 | 108.0-122.6 | 14.6 | Pericentromere | 134.4-135.5 |
| lmk0203 | 1 | 129.4-142.7 | 13.3 | Centromere |  |
| lmk0300 | 1 | 221.4-241.2 | 19.8 |  |  |
| lmk0548 | 2 | 97.3-108.9 | 11.6 | Pericentromere | 92.9-94.6 |
| lmk0556 | 2 | 115.7-125.8 | 10.1 | Pericentromere |  |
| lmk0765 | 3 | 70.0-88.3 | 18.3 | Centromere | 85.5-86.8 |
| lmk0767 | 3 | 91.2-114.7 | 23.5 | Pericentromere |  |
| lmk0979 | 4 | 13.9-28.0 | 14.1 |  | 105.0-106.1 |
| lmk1002 | 4 | 47.8-59.0 | 11.2 |  |  |
| lmk1015 | 4 | 85.7-97.8 | 12.1 | Pericentromere |  |
| lmk1016 | 4 | 97.8-121.5 | 23.7 | Centromere |  |
| lmk1253 | 5 | 99.0-128.9 | 29.9 | Centromere | 102.1-103.7 |
| lmk1431 | 6 | 41.7-58.3 | 16.6 | Centromere | 49.6-50.3 |
| lmk1453 | 6 | 103.0-115.4 | 12.4 |  |  |
| lmk1615 | 7 | 42.4-95.0 | 52.6 | Centromere | 55.0-55.6 |
| lmk1812 | 8 | 35.6-59.0 | 23.4 | Centromere | 49.2-51.0 |
| lmk1815 | 8 | 64.1-104.8 | 40.7 | Pericentromere |  |
| lmk1987 | 9 | 63.2-74.3 | 11.1 | Pericentromere | 51.5-52.6 |
| lmk2044 | 10 | 15.6-70.0 | 54.4 | Centromere | 50.2-51.7 |

^a^ Chr.: number of chromosomes.

**Table S4** Summary of the high-density genetic map derived from two RIL populations

| Pop.^a^ | Chr.^b^ | No. of markers^c^ | Physical length of map (Mb) | Map length (cM) | Average genetic length (cM) | Maximal genetic length (cM) |
| --- | --- | --- | --- | --- | --- | --- |
| HO | 1 | 117 | 300.4 | 262.3 | 2.3 | 14.3 |
|  | 2 | 74 | 236.4 | 184.4 | 2.5 | 16.3 |
|  | 3 | 60 | 217.0 | 186.4 | 3.2 | 15.3 |
|  | 4 | 84 | 238.4 | 145.6 | 1.8 | 9.2 |
|  | 5 | 91 | 208.4 | 176.3 | 2.0 | 15.3 |
|  | 6 | 83 | 167.0 | 150.5 | 1.8 | 14.3 |
|  | 7 | 90 | 175.2 | 180.0 | 2.0 | 11.6 |
|  | 8 | 73 | 164.9 | 142.9 | 2.0 | 12.1 |
|  | 9 | 50 | 148.4 | 128.8 | 2.6 | 12.1 |
|  | 10 | 34 | 138.7 | 84.9 | 2.6 | 13.9 |
|  | Total | 756 | 1994.8 | 1642.2 | 2.2 | 16.3 |
| LR | 1 | 412 | 301.1 | 307.2 | 0.7 | 3.5 |
|  | 2 | 261 | 237.0 | 192.2 | 0.7 | 3.2 |
|  | 3 | 286 | 232.1 | 217.4 | 0.8 | 3.4 |
|  | 4 | 202 | 246.9 | 132.5 | 0.7 | 5.2 |
|  | 5 | 244 | 217.7 | 198.2 | 0.8 | 3.5 |
|  | 6 | 158 | 169.2 | 103.0 | 0.7 | 4.6 |
|  | 7 | 183 | 176.1 | 123.9 | 0.7 | 2.4 |
|  | 8 | 184 | 175.6 | 129.2 | 0.7 | 4.0 |
|  | 9 | 99 | 155.3 | 65.7 | 0.7 | 5.7 |
|  | 10 | 92 | 149.9 | 50.2 | 0.6 | 5.4 |
|  | Total | 2121 | 2060.9 | 1519.5 | 0.7 | 5.7 |

^a^ Pop.: the experimental populations. HO: the high-oil population (B73×BY804); LR: the lodging-resistance population (Zheng58×HD568).

^b^ Chr.: number of chromosomes.

^c^ No. of bins: the number of bin markers on the chromosome.

**Table S5** QTL for rind penetrometer resistance in high-oil population (B73×BY804)

| Env.^a^ | Stage^b^ | Name^c^ | Chr.^d^ | Flanking markers^e^ | Interval (Mb)^f^ | Physical length^g^ (Mb) | LOD^h^ | PVE^i^ | ADD^j^ |
| --- | --- | --- | --- | --- | --- | --- | --- | --- | --- |
| 2012B | V10 | *qAha8* | 8 | hmk647-hmk653 | 127.49-138.39 | 10.90 | 4.57 | 5.74 | 0.04 |
|  | DTS | *qAhb1-1* | 1 | hmk017-hmk020 | 17.74-19.7 | 1.96 | 5.71 | 5.26 | 0.11 |
|  |  | *qAhb1-2* | 1 | hmk093-hmk096 | 242.15-253.03 | 10.88 | 3.98 | 4.40 | -0.10 |
|  |  | *qAhb2* | 2 | hmk123-hmk125 | 22.97-31.29 | 8.33 | 4.94 | 7.44 | -0.13 |
|  |  | *qAhb6* | 6 | hmk456-hmk461 | 99.89-106.34 | 6.46 | 3.59 | 5.11 | 0.11 |
|  |  | *qAhb8* | 8 | hmk606-hmk609 | 5.19-6.59 | 1.40 | 4.27 | 6.85 | -0.13 |
|  | AS10 | *qAhc2* | 2 | hmk123-hmk125 | 22.97-31.29 | 8.33 | 5.54 | 9.15 | -0.22 |
|  |  | *qAhc6-1* | 6 | hmk474-hmk478 | 122.4-131.99 | 9.59 | 3.79 | 6.64 | 0.19 |
|  |  | *qAhc6-2* | 6 | hmk505-hmk508 | 159.62-163.18 | 3.55 | 3.49 | 6.77 | -0.19 |
|  |  | *qAhc9* | 9 | hmk711-hmk713 | 129.19-133.92 | 4.73 | 5.02 | 5.11 | -0.17 |
|  | AS20 | *qAhd2* | 2 | hmk123-hmk125 | 22.97-31.29 | 8.33 | 3.95 | 9.34 | -0.21 |
|  |  | *qAhd6* | 6 | hmk505-hmk508 | 159.62-163.18 | 3.55 | 5.00 | 6.80 | -0.18 |
|  |  | *qAhd9* | 9 | hmk711-hmk713 | 129.19-133.92 | 4.73 | 3.67 | 4.23 | -0.14 |
|  | AS30 | *qAhe1* | 1 | hmk037-hmk041 | 49.39-58.5 | 9.11 | 4.12 | 5.88 | 0.17 |
|  |  | *qAhe2* | 2 | hmk164-hmk168 | 161.05-174.98 | 13.93 | 5.60 | 5.89 | -0.18 |
|  |  | *qAhe6* | 6 | hmk504-hmk506 | 158.47-160.04 | 1.57 | 8.13 | 10.57 | -0.24 |
|  |  | *qAhe7* | 7 | hmk510-hmk511 | 1.3-4.06 | 2.77 | 5.55 | 9.95 | 0.23 |
|  | AS40 | *qAhf1* | 1 | hmk017-hmk020 | 17.74-19.7 | 1.96 | 3.95 | 5.53 | 0.17 |
|  |  | *qAhf6-1* | 6 | hmk477-hmk482 | 129.97-137.9 | 7.92 | 4.51 | 6.06 | 0.18 |
|  |  | *qAhf6-2* | 6 | hmk505-hmk508 | 159.62-163.18 | 3.55 | 4.15 | 7.64 | -0.20 |
|  | AS50 | *qAhg1* | 1 | hmk037-hmk041 | 49.39-58.5 | 9.11 | 6.69 | 7.35 | 0.18 |
|  |  | *qAhg2* | 2 | hmk163-hmk167 | 156.86-173.46 | 16.60 | 5.09 | 5.09 | -0.15 |
|  |  | *qAhg6-1* | 6 | hmk477-hmk482 | 129.97-137.9 | 7.92 | 4.97 | 6.37 | 0.18 |
|  |  | *qAhg6-2* | 6 | hmk504-hmk506 | 158.47-160.04 | 1.57 | 7.50 | 11.58 | -0.24 |
|  |  | *qAhg7* | 7 | hmk510-hmk511 | 1.3-4.06 | 2.77 | 3.56 | 4.90 | 0.15 |
|  |  | *qAhg8* | 8 | hmk638-hmk642 | 113.72-117.76 | 4.04 | 4.43 | 2.15 | -0.10 |
| 2012H | V10 | *qBha9* | 9 | hmk709-hmk710 | 127.82-128.65 | 0.83 | 5.33 | 8.96 | -0.09 |
|  | DTS | *qBhb6* | 6 | hmk505-hmk508 | 159.62-163.18 | 3.55 | 3.69 | 3.57 | -0.09 |
|  |  | *qBhb7* | 7 | hmk579-hmk582 | 153.45-157.9 | 4.45 | 3.60 | 5.43 | -0.11 |
|  | AS10 | *qBhc3* | 3 | hmk229-hmk233 | 175.55-183.25 | 7.70 | 3.96 | 1.95 | 0.09 |
|  |  | *qBhc6-1* | 6 | hmk473-hmk478 | 118.15-131.99 | 13.84 | 6.81 | 8.75 | 0.19 |
|  |  | *qBhc6-2* | 6 | hmk505-hmk508 | 159.62-163.18 | 3.55 | 5.38 | 7.51 | -0.17 |
|  | AS20 | *qBhd6-1* | 6 | hmk474-hmk478 | 122.4-131.99 | 9.59 | 8.31 | 10.33 | 0.23 |
|  |  | *qBhd6-2* | 6 | hmk505-hmk508 | 159.62-163.18 | 3.55 | 5.23 | 7.65 | -0.19 |
|  | AS30 | *qBhe2-1* | 2 | hmk174-hmk177 | 189.64-199.55 | 9.91 | 4.53 | 9.08 | 0.20 |
|  |  | *qBhe2-2* | 2 | hmk184-hmk186 | 219.73-224.56 | 4.83 | 4.01 | 6.14 | -0.16 |
|  |  | *qBhe6* | 6 | hmk505-hmk508 | 159.62-163.18 | 3.55 | 5.85 | 10.17 | -0.19 |
|  |  | *qBhe8* | 8 | hmk609-hmk611 | 6.59-10.86 | 4.27 | 6.98 | 13.61 | -0.22 |
|  | AS40 | *qBhf6-1* | 6 | hmk474-hmk478 | 122.4-131.99 | 9.59 | 4.26 | 8.62 | 0.17 |
|  |  | *qBhf6-2* | 6 | hmk505-hmk508 | 159.62-163.18 | 3.55 | 5.45 | 9.20 | -0.17 |
|  |  | *qBhf8* | 8 | hmk665-hmk669 | 159.39-163 | 3.61 | 3.99 | 3.57 | -0.11 |
|  | AS50 | *qBhg1* | 1 | hmk086-hmk090 | 220.71-234.79 | 14.08 | 4.40 | 2.53 | -0.09 |
|  |  | *qBhg4* | 4 | hmk306-hmk308 | 177.15-181.4 | 4.25 | 4.56 | 8.59 | -0.16 |
|  |  | *qBhg6-1* | 6 | hmk474-hmk478 | 122.4-131.99 | 9.59 | 3.52 | 5.65 | 0.14 |
|  |  | *qBhg6-2* | 6 | hmk506-hmk508 | 160.04-163.18 | 3.14 | 8.49 | 13.31 | -0.21 |
|  |  | *qBhg8* | 8 | hmk626-hmk634 | 72.3-101.41 | 29.11 | 4.63 | 4.68 | -0.12 |
| 2013B | V10 | *qCha1-1* | 1 | hmk091-hmk093 | 235.1-242.15 | 7.05 | 5.58 | 8.40 | -0.05 |
|  |  | *qCha1-2* | 1 | hmk098-hmk104 | 260.15-273.68 | 13.53 | 3.81 | 7.33 | 0.04 |
|  |  | *qCha5* | 5 | hmk347-hmk348 | 25.4-26.56 | 1.16 | 6.21 | 4.03 | -0.03 |
|  | DTS | *qChb1* | 1 | hmk017-hmk020 | 17.74-19.7 | 1.96 | 3.65 | 5.75 | 0.15 |
|  |  | *qChb2* | 2 | hmk123-hmk125 | 22.97-31.29 | 8.33 | 6.04 | 10.26 | -0.21 |
|  |  | *qChb6* | 6 | hmk508-hmk509 | 163.18-167.03 | 3.86 | 3.93 | 6.16 | -0.16 |
|  | AS10 | *qChc1* | 1 | hmk037-hmk041 | 49.39-58.5 | 9.11 | 4.40 | 5.48 | 0.20 |
|  |  | *qChc6* | 6 | hmk508-hmk509 | 163.18-167.03 | 3.86 | 4.14 | 5.38 | -0.20 |
|  | AS20 | *qChd1* | 1 | hmk037-hmk041 | 49.39-58.5 | 9.11 | 7.31 | 8.22 | 0.24 |
|  |  | *qChd3* | 3 | hmk246-hmk250 | 211.97-215.02 | 3.05 | 4.42 | 8.46 | 0.25 |
|  |  | *qChd6* | 6 | hmk505-hmk508 | 159.62-163.18 | 3.55 | 3.78 | 5.32 | -0.19 |
|  |  | *qChd7* | 7 | hmk572-hmk577 | 145.73-151.97 | 6.24 | 4.46 | 5.66 | -0.19 |
|  | AS30 | *qChe1-1* | 1 | hmk037-hmk041 | 49.39-58.5 | 9.11 | 6.06 | 5.62 | 0.19 |
|  |  | *qChe1-2* | 1 | hmk102-hmk106 | 264.52-274.94 | 10.42 | 3.53 | 5.49 | 0.18 |
|  |  | *qChe3* | 3 | hmk245-hmk250 | 210.76-215.02 | 4.26 | 6.65 | 12.25 | 0.29 |
|  |  | *qChe6* | 6 | hmk508-hmk509 | 163.18-167.03 | 3.86 | 3.66 | 5.23 | -0.18 |
|  | AS40 | *qChf1* | 1 | hmk102-hmk109 | 264.52-276.99 | 12.47 | 5.14 | 6.24 | 0.19 |
|  |  | *qChf3* | 3 | hmk246-hmk250 | 211.97-215.02 | 3.05 | 4.26 | 13.44 | 0.29 |
|  | AS50 | *qChg1* | 1 | hmk017-hmk020 | 17.74-19.7 | 1.96 | 3.46 | 4.60 | 0.16 |
|  |  | *qChg6* | 6 | hmk505-hmk508 | 159.62-163.18 | 3.55 | 3.90 | 8.12 | -0.21 |

^a^ Env.: the location of field experiments. 2012B: Beijing in 2012; 2012H: Hainan in 2012; 2013B: Beijing in 2013.

^b^ The investigation stage of rind penetrometer resistance. V10: the tenth-leaf stage; DTS: days to silking; AS10: ten days after silking; AS20: twenty days after silking; AS30: thirty days after silking; AS40: forty days after silking; AS50: fifty days after silking.

^c^ The name of each QTL consists of the information of environments (A for 2012B; B for 2012H; C for 2013B), stage (a for V10; b for DTS; c for AS10; d for AS20; e for AS30; f for AS40; g for AS50), population type (h for high-oil population; l for lodging-resistance population), and the number of chromosomes.

^d^ Chr.: number of chromosomes.

^e^ Flanking markers: the marginal markers of confidence interval of each QTL.

^f^ Interval: physical range of flanking markers.

^g^ Physical length: physical distance between flanking markers.

^h^ LOD: the likelihood of odds.

^I^ PVE: the phenotypic variance explained by individual QTL.

^j^ ADD: the additive effect value.

**Table S6** QTL for rind penetrometer resistance in lodging-resistance population (Zheng58×HD568)

| Env.^a^ | Stage^b^ | Name^c^ | Chr.^d^ | Flanking markers^e^ | Interval (Mb)^f^ | Physical length^g^ (Mb) | LOD^h^ | PVE^i^ | ADD^j^ |
| --- | --- | --- | --- | --- | --- | --- | --- | --- | --- |
| 2012H | V10 | *qBla4* | 4 | lmk1071-lmk1087 | 179.66-185.47 | 5.81 | 3.71 | 8.49 | -0.11 |
|  |  | *qBla7* | 7 | lmk1614-lmk1622 | 42.23-106.72 | 64.49 | 5.81 | 11.55 | 0.13 |
|  |  | *qBla8* | 8 | lmk1900-lmk1909 | 163.65-164.87 | 1.21 | 6.19 | 7.19 | -0.10 |
|  | DTS | *qBlb3* | 3 | lmk861-lmk865 | 184.76-187.18 | 2.42 | 3.73 | 1.85 | 0.06 |
|  |  | *qBlb4-1* | 4 | lmk968-lmk976 | 8.9-10.77 | 1.87 | 3.69 | 4.51 | -0.09 |
|  |  | *qBlb4-2* | 4 | lmk1081-lmk1097 | 181.71-189.04 | 7.33 | 6.40 | 7.58 | -0.12 |
|  |  | *qBlb5* | 5 | lmk1285-lmk1290 | 168.96-173.41 | 4.45 | 3.71 | 5.15 | 0.10 |
|  |  | *qBlb8* | 8 | lmk1830-lmk1847 | 120.1-140.75 | 20.65 | 6.52 | 8.68 | 0.13 |
|  |  | *qBlb10* | 10 | lmk2075-lmk2081 | 123.63-136.91 | 13.29 | 5.43 | 5.03 | -0.10 |
|  | AS10 | *qBlc3* | 3 | lmk689-lmk696 | 9.93-12.88 | 2.94 | 4.02 | 4.24 | -0.11 |
|  |  | *qBlc4* | 4 | lmk968-lmk977 | 8.9-12.03 | 3.13 | 4.45 | 7.67 | -0.14 |
|  |  | *qBlc8* | 8 | lmk1831-lmk1840 | 120.46-130.27 | 9.81 | 10.53 | 14.06 | 0.20 |
|  | AS20 | *qBld1* | 1 | lmk217-lmk223 | 167.21-180.12 | 12.91 | 4.21 | 3.01 | 0.08 |
|  |  | *qBld8* | 8 | lmk1833-lmk1847 | 120.8-140.75 | 19.95 | 5.15 | 8.56 | 0.14 |
|  | AS30 | *qBle4* | 4 | lmk1098-lmk1104 | 189.04-196.94 | 7.90 | 4.26 | 6.88 | -0.11 |
|  |  | *qBle8-1* | 8 | lmk1776-lmk1781 | 12.27-15.14 | 2.87 | 4.13 | 4.80 | 0.09 |
|  |  | *qBle8-2* | 8 | lmk1831-lmk1838 | 120.46-126.49 | 6.03 | 10.05 | 7.97 | 0.12 |
|  | AS40 | *qBlf1-1* | 1 | lmk283-lmk293 | 212.31-217.42 | 5.11 | 5.77 | 6.65 | 0.11 |
|  |  | *qBlf1-2* | 1 | lmk389-lmk394 | 289.55-290.68 | 1.14 | 3.92 | 2.43 | -0.06 |
|  |  | *qBlf4* | 4 | lmk1071-lmk1088 | 179.66-185.64 | 5.98 | 3.87 | 5.58 | -0.10 |
|  | AS50 | *qBlg1* | 1 | lmk266-lmk274 | 203.36-207.13 | 3.77 | 3.97 | 3.41 | 0.08 |
|  |  | *qBlg6* | 6 | lmk1437-lmk1441 | 67.84-76.1 | 8.26 | 4.55 | 2.91 | 0.07 |
|  |  | *qBlg8* | 8 | lmk1831-lmk1840 | 120.46-130.27 | 9.81 | 5.55 | 7.72 | 0.12 |
| 2013B | V10 | *qCla1* | 1 | lmk287-lmk298 | 213.54-221.24 | 7.70 | 5.73 | 7.54 | -0.03 |
|  |  | *qCla7-1* | 7 | lmk1575-lmk1580 | 11.83-14.61 | 2.79 | 5.28 | 7.86 | 0.03 |
|  |  | *qCla7-2* | 7 | lmk1690-lmk1696 | 152.95-157.76 | 4.81 | 3.81 | 5.23 | 0.03 |
|  | DTS | *qClb3-1* | 3 | lmk674-lmk682 | 0.47-8.62 | 8.15 | 4.73 | 2.21 | 0.09 |
|  |  | *qClb3-2* | 3 | lmk945-lmk953 | 224.57-225.51 | 0.94 | 3.90 | 5.35 | -0.14 |
|  |  | *qClb4* | 4 | lmk1071-lmk1084 | 179.66-184.83 | 5.17 | 3.74 | 7.08 | -0.16 |
|  |  | *qClb5* | 5 | lmk1378-lmk1383 | 208.1-209.57 | 1.47 | 4.69 | 5.97 | 0.15 |
|  | AS10 | *qClc5* | 5 | lmk1379-lmk1386 | 208.49-210.4 | 1.91 | 4.03 | 3.28 | 0.14 |
|  |  | *qClc8* | 8 | lmk1831-lmk1841 | 120.46-130.39 | 9.93 | 6.50 | 11.03 | 0.26 |
|  | AS20 | *qCld4* | 4 | lmk968-lmk977 | 8.9-12.03 | 3.13 | 4.15 | 3.69 | -0.15 |
|  |  | *qCld5-1* | 5 | lmk1211-lmk1220 | 28.02-36.41 | 8.40 | 4.43 | 5.47 | 0.18 |
|  |  | *qCld5-2* | 5 | lmk1379-lmk1383 | 208.49-209.57 | 1.08 | 4.00 | 2.35 | 0.12 |
|  | AS30 | *qCle4* | 4 | lmk970-lmk975 | 10.07-10.77 | 0.70 | 7.25 | 6.79 | -0.19 |
|  |  | *qCle5* | 5 | lmk1227-lmk1234 | 46.08-69.55 | 23.47 | 6.30 | 8.71 | 0.22 |
|  |  | *qCle8* | 8 | lmk1831-lmk1838 | 120.46-126.49 | 6.03 | 8.35 | 7.98 | 0.21 |
|  | AS40 | *qClf3* | 3 | lmk943-lmk948 | 224.13-224.9 | 0.78 | 5.88 | 7.33 | -0.17 |
|  |  | *qClf5-1* | 5 | lmk1227-lmk1237 | 46.08-71.86 | 25.78 | 4.81 | 5.45 | 0.15 |
|  |  | *qClf5-2* | 5 | lmk1378-lmk1384 | 208.1-209.87 | 1.77 | 4.51 | 4.33 | 0.14 |
|  |  | *qClf8* | 8 | lmk1826-lmk1841 | 118.33-130.39 | 12.06 | 5.60 | 8.64 | 0.19 |
|  | AS50 | *qClg3* | 3 | lmk943-lmk948 | 224.13-224.9 | 0.78 | 3.50 | 4.30 | -0.14 |
|  |  | *qClg5* | 5 | lmk1227-lmk1234 | 46.08-69.55 | 23.47 | 5.46 | 9.63 | 0.20 |
|  |  | *qClg8* | 8 | lmk1790-lmk1799 | 18.73-23.77 | 5.05 | 4.53 | 7.72 | 0.18 |

^a^ Env.: the location of field experiments. 2012H: Hainan in 2012; 2013B: Beijing in 2013.

^b^ The investigation stage of rind penetrometer resistance. V10: the tenth-leaf stage; DTA: days to silking; AF10: ten days after silking; AF20: twenty days after silking; AF30: thirty days after silking; AF40: forty days after silking; AF50: fifty days after silking.

^c^ The name of each QTL consists of the information of environments (B for 2012H; C for 2013B), stage (a for V10; b for DTS; c for AS10; d for AS20; e for AS30; f for AS40; g for AS50), population type (h for high-oil population; l for lodging-resistance population), and the number of chromosomes.

^d^ Chr.: number of chromosomes.

^e^ Flanking markers: the marginal markers of confidence interval of each QTL.

^f^ Interval: physical range of flanking markers.

^g^ Physical length: physical distance between flanking markers.

^h^ LOD: the likelihood of odds.

^I^ PVE: the phenotypic variance explained by individual QTL.

^j^ ADD: the additive effect value.

**Table S7** Candidate genes annotation

| Chr.^a^ | Gene model ID | Pos. start^b^ | Pos. end^c^ | Annotation |
| --- | --- | --- | --- | --- |
| 1 | GRMZM2G124532 | 50034523 | 50023180 | phytochromeB1 |
| 1 | GRMZM2G152891 | 52096639 | 52092838 | calmodulin3 |
| 1 | GRMZM2G018728 | 52807763 | 52805139 | protein kinase inhibitor1 |
| 1 | GRMZM2G107597 | 57251591 | 57248463 | sugars will eventually be exported transporter16 |
| 1 | GRMZM2G019434 | 171558256 | 171553382 | MAP kinase kinase kinase51 |
| 1 | GRMZM2G171822 | 173867297 | 173865554 | barren inflorescence2 |
| 1 | GRMZM2G402631 | 178036072 | 178035095 | pathogenesis related protein5 |
| 1 | GRMZM2G080603 | 178505233 | 178503667 | glycine-rich protein1 |
| 1 | GRMZM2G056400 | 215662587 | 215657189 | KANADI1 |
| 1 | GRMZM2G347280 | 217836445 | 217833921 | trehalose-6-phosphate phosphatase1 |
| 1 | GRMZM2G008226 | 218623790 | 218618199 | trehalose-6-phosphate synthase4 |
| 1 | GRMZM5G869779 | 226070426 | 226063076 | folylpolyglutamate synthetase2: Paralog of bm4 also encoding FPGS |
| 1 | GRMZM2G415359 | 231403913 | 231398148 | malate dehydrogenase4 |
| 1 | GRMZM2G011357 | 239671192 | 239667869 | indeterminate growth1 |
| 1 | GRMZM2G081554 | 241285679 | 241277428 | anther ear1 |
| 1 | GRMZM2G016241 | 241432121 | 241430768 | bronze2: like bz1 |
| 1 | GRMZM2G083248 | 243277640 | 243274177 | sucrose transporter GRMZM2G083248: |
| 1 | GRMZM2G167986 | 243709449 | 243707108 | cytochrome P-450 8 |
| 1 | GRMZM2G457370 | 250137737 | 250132189 | argonaute18b |
| 1 | GRMZM2G364069 | 250156813 | 250153164 | chaperone DNA J2 |
| 1 | GRMZM2G014392 | 250956063 | 250953388 | viviparous14 |
| 1 | GRMZM5G858417 | 263068441 | 263063356 | leaf permease1 |
| 1 | GRMZM2G144744 | 266163168 | 266160101 | dwarf plant8 |
| 1 | GRMZM2G024104 | 267963551 | 267960169 | glutamine synthetase2 |
| 1 | GRMZM2G442658 | 274054148 | 274050254 | alcohol dehydrogenase1 |
| 1 | GRMZM2G159295 | 275058949 | 275052382 | CorA-like magnesium transporter protein putative expressed |
| 1 | GRMZM2G111642 | 290672751 | 290666783 | cellulose synthase5: |
| 2 | GRMZM2G177812 | 24503843 | 24498593 | ABC transporter G family member 11 |
| 2 | GRMZM2G178693 | 28495467 | 28493204 | plasma membrane intrinsic protein2 |
| 2 | GRMZM2G118950 | 28940284 | 28938473 | ammonium transporter3 |
| 2 | GRMZM2G137751 | 30023024 | 30021407 | calcineurin B-like12 |
| 2 | GRMZM2G018241 | 161763704 | 161757546 | cellulose synthase9: cDNA |
| 2 | GRMZM2G092125 | 169190423 | 169186912 | plasma membrane intrinsic protein2 |
| 2 | GRMZM2G145944 | 171519145 | 171514014 | phospholipase D12 |
| 2 | GRMZM2G108912 | 197194172 | 197187325 | phospholipase D10 |
| 2 | GRMZM2G094955 | 223377937 | 223375329 | sugars will eventually be exported transporter14a |
| 2 | GRMZM2G151227 | 224561990 | 224558005 | white pollen1 |
| 3 | GRMZM2G060974 | 2957584 | 2955821 | sugars will eventually be exported transporter3b |
| 3 | GRMZM2G102770 | 3429262 | 3428861 | expressed protein |
| 3 | GRMZM2G132116 | 6797402 | 6793085 | aurora b kinase1 |
| 3 | GRMZM2G146644 | 7605870 | 7601132 | cytokinin oxidase1 |
| 3 | GRMZM2G118243 | 10286585 | 10276457 | glossy13 |
| 3 | GRMZM2G036340 | 10457618 | 10455337 | dwarf plant1 |
| 3 | GRMZM2G039454 | 11655958 | 11648913 | cellulose synthase3: cDNA |
| 3 | GRMZM2G002276 | 179842136 | 179837079 | transcription associated factor1 |
| 3 | GRMZM2G059453 | 180098099 | 180096139 | protein phosphatase homolog3 |
| 3 | GRMZM2G050089 | 185817191 | 185812392 | auxin efflux carrier component putative expressed |
| 4 | GRMZM2G109140 | 180229331 | 180232615 | fbl41 - F-box protein41 |
| 4 | GRMZM2G162434 | 185830259 | 185828677 | glossy3: like gl1, but surface wax has all rice-grain-type particles |
| 4 | GRMZM2G422750 | 192761788 | 192758391 | colorless2 |
| 4 | Zm00001d052598 | 194315585 | 194314663 | narrow sheath2: duplicate factor with ns1 |
| 4 | AC225127.3_FG003 | 195239581 | 195238767 | male sterile44: dominant Ms44 plants male sterile |
| 5 | GRMZM2G154954 | 31465552 | 31464367 | glycine-rich protein3: single or low copy number cDNA |
| 5 | GRMZM2G088212 | 63597617 | 63593142 | catalase1 |
| 5 | GRMZM2G361518 | 64796881 | 64791077 | argonaute1d: Ortholog of Arabidopsis ago1 |
| 5 | GRMZM2G057000 | 65143833 | 65138723 | nana plant2: like na1 |
| 5 | GRMZM2G163849 | 68913355 | 68902393 | brahma1 |
| 5 | GRMZM2G114793 | 69357915 | 69354043 | Binding protein homolog1 |
| 5 | GRMZM2G139300 | 169502089 | 169497597 | cell wall invertase1 |
| 5 | GRMZM5G868908 | 169727140 | 169710822 | rar1 putative expressed |
| 6 | GRMZM2G079080 | 103293200 | 103286236 | argonaute10b |
| 6 | GRMZM2G122871 | 104235309 | 104230611 | elongation factor gamma1 |
| 6 | GRMZM2G064657 | 122582074 | 122577593 | phosphate transporter4 |
| 6 | GRMZM2G135108 | 125194486 | 125196087 | guaiacol peroxidase3: |
| 6 | GRMZM2G027723 | 128743576 | 128750030 | cesa2 - cellulose synthase2 |
| 6 | GRMZM2G027723 | 128750030 | 128743576 | cellulose synthase2: cDNA |
| 6 | GRMZM2G141810 | 129847591 | 129844054 | alliin lyase precursor putative expressed |
| 6 | GRMZM5G864847 | 130190406 | 130188997 | barren inflorescence4 |
| 6 | GRMZM2G052890 | 132016861 | 132008587 | Zea AGAMOUS homolog1 |
| 6 | GRMZM2G328785 | 133826469 | 133823627 | protein kinase1 |
| 6 | GRMZM2G046686 | 136151875 | 136146698 | hexokinase8 |
| 6 | GRMZM2G079440 | 137333893 | 137332062 | dehydrin1 |
| 6 | AC233866.1_FG006 | 137603647 | 137601820 | elongation factor alpha1 |
| 6 | GRMZM2G001221 | 159458229 | 159455359 | calcineurin B-like2 |
| 6 | GRMZM2G074361 | 160358354 | 160357192 | profilin homolog1 |
| 6 | GRMZM2G320373 | 162860116 | 162859289 | lipid transfer protein1 |
| 6 | GRMZM2G031200 | 164697457 | 164699173 | nactf87 - NAC-transcription factor 87 |
| 6 | GRMZM2G154595 | 165836297 | 165831537 | malate dehydrogenase2 |
| 7 | GRMZM2G092604 | 79123848 | 79119386 | bri1-like receptor kinase1 |
| 7 | GRMZM2G134797 | 149954818 | 149952414 | nucleotide diphosphate kinase1: leaf cDNA csu269, single copy |
| 8 | GRMZM2G416836 | 5500321 | 5488159 | trehalose-6-phosphate synthase14 |
| 8 | GRMZM2G034157 | 21621116 | 21620266 | heat shock protein18c |
| 8 | GRMZM2G119809 | 72907132 | 72904843 | acidic ribosomal protein P2a |
| 8 | GRMZM2G097768 | 73424707 | 73418620 | sugar transport1 |
| 8 | GRMZM2G383807 | 77504702 | 77501631 | protein phosphatase homolog15 |
| 8 | GRMZM2G112336 | 79744724 | 79738607 | cellulose synthase1 |
| 8 | GRMZM2G375504 | 95237335 | 95226658 | proline responding1 |
| 8 | GRMZM2G126010 | 99909909 | 99906105 | actin1 |
| 8 | GRMZM2G068943 | 123138783 | 123130508 | trehalose-6-phosphate synthase1 |
| 8 | GRMZM2G056014 | 132413070 | 132408451 | calcium pump1 |
| 8 | GRMZM2G028393 | 133997534 | 133996633 | subtilisin-chymotrypsin inhibitor homolog1 |
| 8 | GRMZM2G420119 | 139129894 | 139126558 | mitochrondrial carrier family protein2 |
| 8 | GRMZM5G875238 | 161471194 | 161465833 | sucrose phosphate synthase1 |
| 8 | GRMZM2G053898 | 161573399 | 161569233 | ubiquitin family domain containing protein expressed |
| 8 | GRMZM2G082390 | 161724576 | 161721257 | ubiquitin family domain containing protein expressed |
| 8 | GRMZM2G065971 | 162842987 | 162836382 | magnesium transporter8 |
| 8 | GRMZM2G049418 | 164490694 | 164487836 | gibberellin 20-oxidase5 |
| 8 | GRMZM2G316362 | 164733879 | 164731500 | stearoyl-acyl-carrier-protein desaturase9 |
| 9 | GRMZM5G844723 | 129489588 | 129484814 | high-sulfur keratin homolog1 |
| 9 | GRMZM2G058522 | 129538722 | 129535000 | superoxide dismutase9 |
| 9 | GRMZM2G099609 | 130030345 | 130027643 | sugars will eventually be exported transporter12b |
| 9 | GRMZM2G067546 | 131431860 | 131423638 | vacuolar sorting receptor homolog1 |
| 10 | GRMZM2G175140 | 130103747 | 130101668 | ammonium transporter1 |
| 10 | GRMZM2G139797 | 130168203 | 130166275 | caleosin related protein putative expressed |
| 10 | GRMZM2G005353 | 133136401 | 133133767 | yabby14 |
| 10 | AC235534.1_FG007 | 136964001 | 136957427 | outer cell layer2 |

^a^ Chr.: number of chromosomes.

^b^ Pos. start: the start position of the candidate gene.

^c^ Pos. end: the end position of the candidate gene.

**Table S8** Comparison of prediction accuracies between models

| Pop.^a^ | Env.^b^ | Model^c^ | V10^d^ | DTS | AS10 | AS20 | AS30 | AS40 | AS50 |
| --- | --- | --- | --- | --- | --- | --- | --- | --- | --- |
| HO | 2012B | UV | 0.20(0.14) | 0.38(0.12) | 0.37(0.13) | 0.46(0.11) | 0.44(0.12) | 0.38(0.12) | 0.44(0.11) |
|  |  | FIXED | **0.26**(0.13) | **0.52**(0.11) | **0.45**(0.12) | **0.52**(0.10) | **0.58**(0.08) | **0.48**(0.11) | **0.58**(0.09) |
|  | 2012H | UV | 0.06(0.15) | 0.31(0.14) | 0.34(0.13) | 0.44(0.11) | 0.38(0.13) | 0.31(0.13) | 0.45(0.12) |
|  |  | FIXED | **0.27**(0.13) | **0.41**(0.12) | **0.44**(0.13) | **0.48**(0.11) | **0.54**(0.11) | **0.44**(0.13) | **0.56**(0.10) |
|  | 2013B | UV | 0.26(0.13) | 0.33(0.14) | 0.35(0.12) | 0.52(0.11) | 0.49(0.11) | 0.48(0.13) | 0.37(0.13) |
|  |  | FIXED | **0.38**(0.12) | **0.46**(0.12) | **0.44**(0.14) | **0.63**(0.09) | **0.58**(0.12) | **0.52**(0.11) | **0.46**(0.11) |
| LR | 2012H | UV | 0.54(0.09) | 0.49(0.12) | 0.43(0.12) | 0.39(0.10) | 0.52(0.11) | 0.38(0.12) | 0.41(0.11) |
|  |  | FIXED | **0.57**(0.08) | **0.58**(0.10) | **0.52**(0.10) | **0.41**(0.12) | **0.57**(0.10) | **0.44**(0.10) | **0.47**(0.10) |
|  | 2013B | UV | 0.48(0.10) | 0.45(0.10) | 0.49(0.10) | 0.50(0.09) | 0.58(0.09) | 0.57(0.10) | 0.52(0.09) |
|  |  | FIXED | **0.53**(0.10) | **0.49**(0.09) | **0.49**(0.10) | **0.52**(0.10) | **0.61**(0.08) | **0.61**(0.08) | **0.53**(0.11) |

^a^ Pop.: the experimental populations. HO: the high-oil population (B73×BY804); LR: the lodging-resistance population (Zheng58×HD568).

^b^ Env.: the location of field experiments. 2012B: Beijing in 2012; 2012H: Hainan in 2012; 2013B: Beijing in 2013.

^c^ UV: univariate model (the general GBLUP); FIXED: the GBLUP model considering RPR-relevant QTL as fixed effects.

^d^ The investigation stage of rind penetrometer resistance. V10: the tenth-leaf stage; DTS: days to silking; AS10: ten days after silking; AS20: twenty days after silking; AS30: thirty days after silking; AS40: forty days after silking; AS50: fifty days after silking. The number in parentheses is standard deviation.

**Table S9** Proportion of variance components estimated by UV, FIXED and ME models

| Model^a^ | Stage^b^ | $\text{σ}_{\text{β}}^{\text{2}}$^c^ | $\text{σ}_{\text{u}}^{\text{2}}$ | $\text{σ}_{\text{v}}^{\text{2}}$ | $\text{σ}_{\text{ε}}^{\text{2}}$ |
| --- | --- | --- | --- | --- | --- |
| UV | V10 |  | 0.13(0.03) |  | 0.87(0.03) |
|  | DTS |  | 0.18(0.04) |  | 0.82(0.04) |
|  | AS10 |  | 0.20(0.04) |  | 0.80(0.04) |
|  | AS20 |  | 0.34(0.05) |  | 0.66(0.05) |
|  | AS30 |  | 0.32(0.05) |  | 0.68(0.05) |
|  | AS40 |  | 0.29(0.05) |  | 0.71(0.05) |
|  | AS50 |  | 0.20(0.04) |  | 0.80(0.04) |
| FIXED | V10 | 0.21(0.05) | 0.08(0.02) |  | 0.71(0.05) |
|  | DTS | 0.25(0.07) | 0.07(0.02) |  | 0.68(0.06) |
|  | AS10 | 0.21(0.07) | 0.14(0.03) |  | 0.65(0.07) |
|  | AS20 | 0.39(0.04) | 0.12(0.02) |  | 0.49(0.03) |
|  | AS30 | 0.37(0.03) | 0.09(0.02) |  | 0.54(0.04) |
|  | AS40 | 0.29(0.05) | 0.12(0.03) |  | 0.59(0.06) |
|  | AS50 | 0.22(0.06) | 0.11(0.02) |  | 0.68(0.06) |
| ME | V10 |  | 0.07(0.02) | 0.24(0.05) | 0.69(0.05) |
|  | DTS |  | 0.06(0.02) | 0.43(0.04) | 0.51(0.04) |
|  | AS10 |  | 0.06(0.01) | 0.41(0.05) | 0.53(0.05) |
|  | AS20 |  | 0.11(0.02) | 0.46(0.04) | 0.43(0.04) |
|  | AS30 |  | 0.10(0.02) | 0.45(0.03) | 0.45(0.03) |
|  | AS40 |  | 0.11(0.03) | 0.39(0.06) | 0.50(0.05) |
|  | AS50 |  | 0.05(0.01) | 0.37(0.06) | 0.58(0.06) |

^a^ UV: univariate model (the general GBLUP); FIXED: the GBLUP model considering RPR-relevant QTL as fixed effects; ME: multivariate GBLUP model using phenotypic data evaluated in other environments as auxiliary variate.

^b^ The investigation stage of rind penetrometer resistance. V10: the tenth-leaf stage; DTS: days to silking; AS10: ten days after silking; AS20: twenty days after silking; AS30: thirty days after silking; AS40: forty days after silking; AS50: fifty days after silking.

^c^ $\text{σ}_{\text{β}}^{\text{2}}$: variance component corresponding to fixed effects; $\text{σ}_{\text{u}}^{\text{2}}$: additive genetic variance; $\text{σ}_{\text{v}}^{\text{2}}$: variance component corresponding to auxiliary variates; $\text{σ}_{\text{ε}}^{\text{2}}$: residual variance.

**Table S10** Proportion of variance components estimated by UV and MS models

| Pop.^a^ | Env.^b^ | UV^c^ | | MS | | |
| --- | --- | --- | --- | --- | --- | --- |
|  |  | $\text{σ}_{\text{u}}^{\text{2}}$ ^d^ | $\text{σ}_{\text{ε}}^{\text{2}}$ | $\text{σ}_{\text{u}}^{\text{2}}$ | $\text{σ}_{\text{v}}^{\text{2}}$ | $\text{σ}_{\text{ε}}^{\text{2}}$ |
| HO | 2012B | 0.25(0.04) | 0.75(0.04) | 0.02(0.01) | 0.80(0.01) | 0.18(0.01) |
|  | 2012H | 0.26(0.04) | 0.74(0.04) | 0.03(0.01) | 0.62(0.04) | 0.35(0.04) |
|  | 2013B | 0.20(0.04) | 0.80(0.04) | 0.05(0.01) | 0.64(0.03) | 0.31(0.02) |
| LR | 2012H | 0.22(0.03) | 0.78(0.03) | 0.04(0.01) | 0.71(0.02) | 0.25(0.02) |
|  | 2013B | 0.32(0.03) | 0.68(0.03) | 0.03(0.01) | 0.51(0.03) | 0.46(0.03) |

^a^ Pop.: the experimental populations. HO: the high-oil population (B73×BY804); LR: the lodging-resistance population (Zheng58×HD568).

^b^ Env.: the location of field experiments. 2012B: Beijing in 2012; 2012H: Hainan in 2012; 2013B: Beijing in 2013.

^c^ UV: univariate model (the general GBLUP); MS: multivariate GBLUP model using phenotypic data evaluated in other stages as auxiliary variate.

^d^ $\text{σ}_{\text{u}}^{\text{2}}$: additive genetic variance; $\text{σ}_{\text{v}}^{\text{2}}$: variance component corresponding to auxiliary variates; $\text{σ}_{\text{ε}}^{\text{2}}$: residual variance.


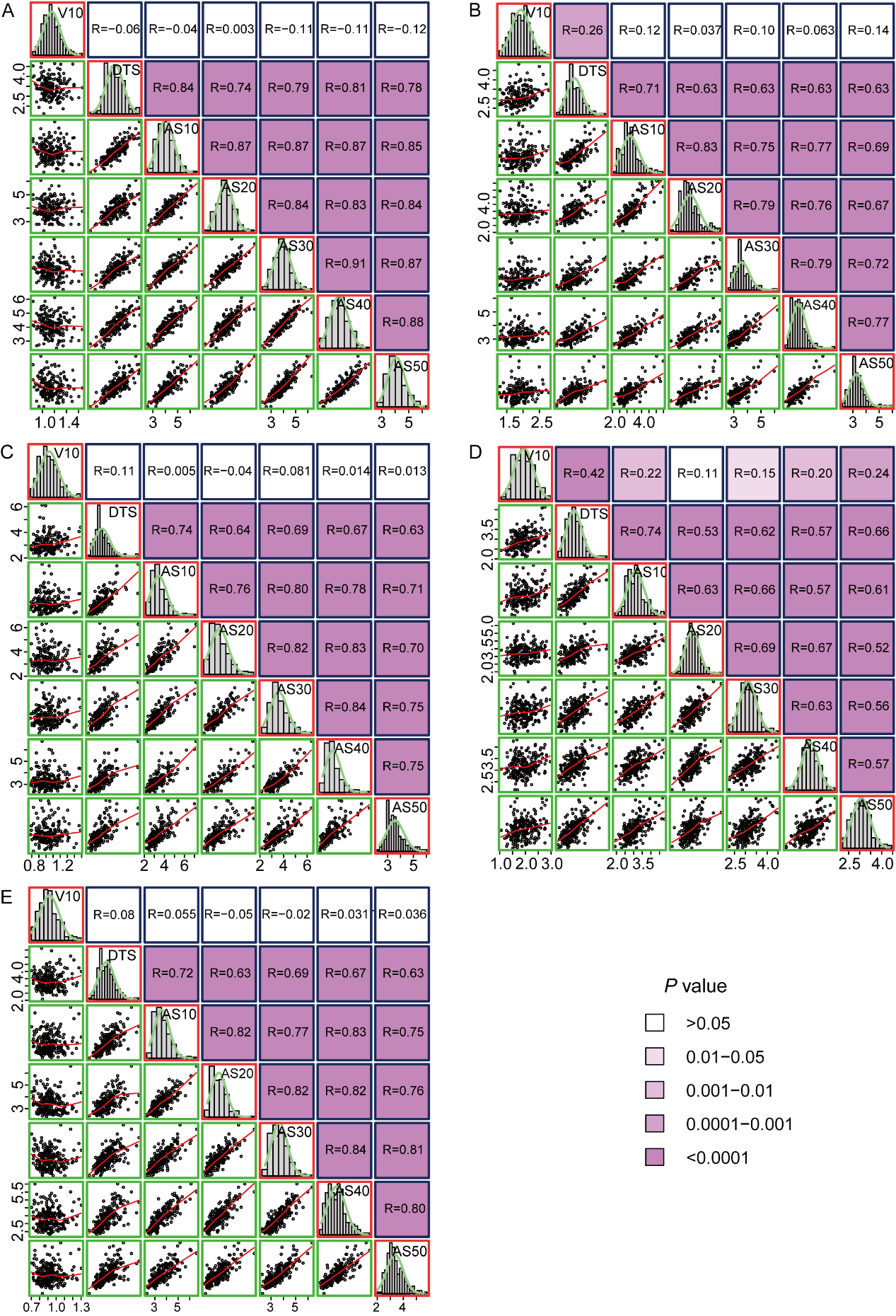


**Figure S1** Phenotypic correlation of rind penetrometer resistance between all pairs of stages within each environment in two RIL populations. (A) to (C) High-oil population (B73×BY804) in Beijing in 2012, Hainan in 2012 and Beijing in 2013. (D) to (E) Lodging-resistance population (Zheng58×HD568) in Hainan in 2012 and Beijing in 2013. V10: the tenth-leaf stage; DTS: days to silking; AS10: ten days after silking; AS20: twenty days after silking; AF30: thirty days after silking; AS40: forty days after silking; AS50: fifty days after silking.


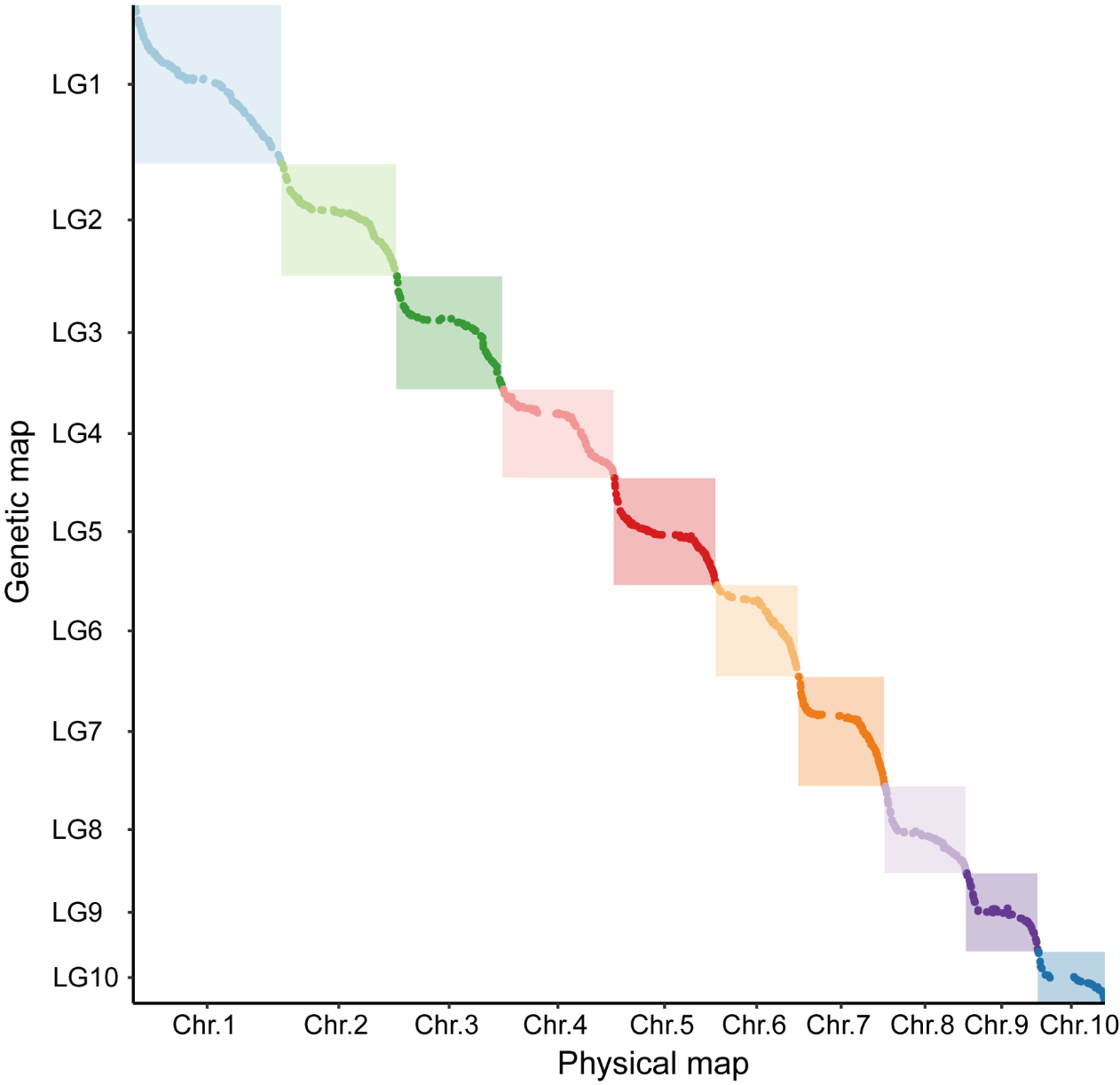


**Figure S2** Comparison of the physical map and genetic map constructed with bin markers in the high-oil population (B73×BY804). The x-axis refers to the linear order of bins based on physical positions in the maize reference genome, and the y-axis denotes the order of bins based on genetic distance in the linkage map; LG: linkage group; Chr.: chromosome.


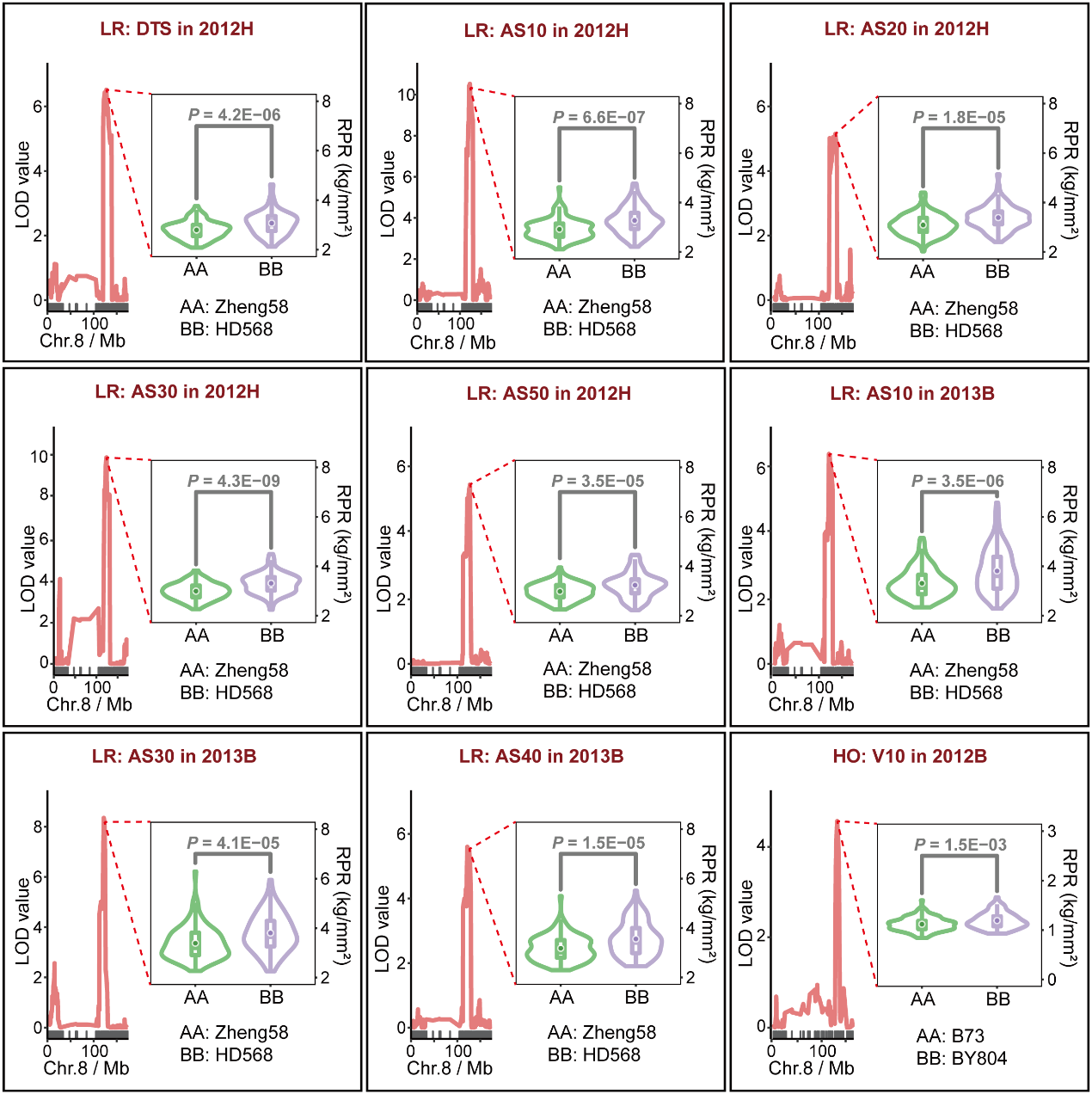


**Figure S3** Illustration of pQTL8 identified in various situations. Violin plots denote the difference between genotypes derived from each parent; HO: the high-oil population (B73×BY804); LR: the lodging-resistance population (Zheng58×HD568); RPR: rind penetrometer resistance; V10: the tenth-leaf stage; DTS: days to silking; AS10: ten days after silking; AS20: twenty days after silking; AF30: thirty days after silking; AS40: forty days after silking; AS50: fifty days after silking; 2012H: Hainan in 2012; 2013B: Beijing in 2013.
